# Structural and Dynamical Response of Lipid Bilayers to Solvation of an Amphiphilic Anesthetic

**DOI:** 10.1021/acs.jpcb.4c05176

**Published:** 2025-01-24

**Authors:** Adriana Šturcová

**Affiliations:** Department of Polymers for Electronics and Photonics, Institute of Macromolecular Chemistry, Czech Academy of Sciences, Heyrovského nám. 2, Prague 6 162 00, Czech Republic

## Abstract

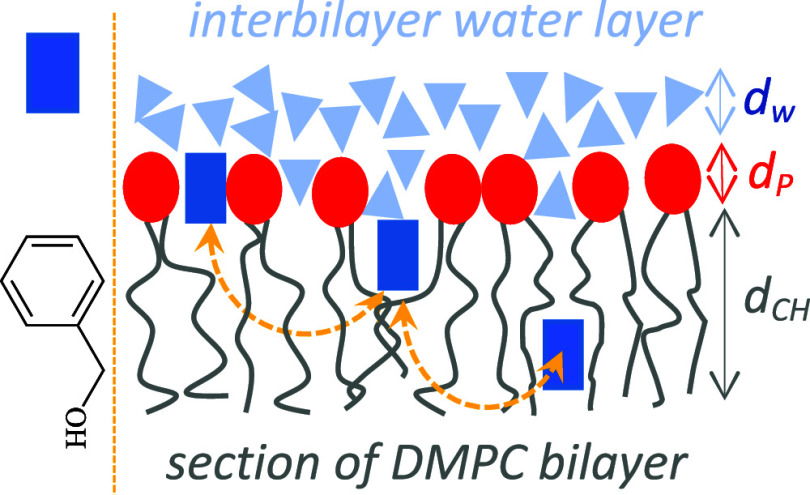

The structural response of 1,2-dimyristoyl-*sn-*glycero-3-phosphatidylcholine (DMPC)/water bilayers to addition and subsequent solvation of a small amphiphilic molecule – an anesthetic benzyl alcohol – was studied by means of solid-state NMR (^2^H NMR, ^31^P NMR) spectroscopy and low-angle X-ray diffraction. The sites of binding of this solute molecule within the bilayer were determined – the solute was shown to partition between several sites in the bilayer and the equilibrium was shown to be dynamic and dependent on the level of hydration and temperature. At the same time, it was shown that solubilization of benzyl alcohol reached a solubility limit and was terminated when the ordering profile of DMPC hydrocarbon chains adopted finite limiting values throughout the whole chain. Such findings were made probably for the first time for any lipid bilayer system and possibly have more general implications for dissolution of other small-molecule amphiphilic solutes in lipid bilayer systems other than DMPC. The limit to the hydrocarbon chain profile is probably a more general property and corresponds to the balance of intrabilayer and interbilayer forces established in combination with the elastic properties of the bilayer system that still consists of one single phase just before the solute forms an excess phase. It is not necessary to quantify the contribution of each individual intrabilayer and interbilayer force acting within such a bilayer system. A model of the dependence of surface density of lipid chains on the chain segment order parameter was also developed – an empirical mathematical model based on experimental data was derived and it was proposed to represent a relationship between intrinsic bilayer forces and bilayer deformation characteristics and might be proven to be of more general significance in the future.

## Introduction

### Solvation and Binding of Benzyl Alcohol Solute by DMPC Bilayers

Lipid bilayers serve as model systems for biomembranes that form boundaries between the exterior and the interior of cells or cellular organelles. The function of biomembranes or bilayers is dependent on their physical properties and can be regulated by molecules dissolved within the bilayers—*e.g.*, sterols, alcohols.^1^ The effect of any solute on a bilayer depends on where it is localized within the bilayer.

Benzyl alcohol is produced by plants and is also synthesized on an industrial scale. It can serve as local anesthetic and is also used in cosmetics and medical formulations due to its bacteriostatic and antifungal properties.

In order to assess the interaction of benzyl alcohol with the lipid bilayer, it is essential to identify its binding sites and its distribution between these sites and to study factors that affect the interaction. In the studies so far, the following sites for benzyl alcohol accommodation have been proposed to exist in amphiphilic bilayers: (1) Headgroup sites. Localization of benzyl alcohol in the polar headgroup region has been suggested by Colley and Metcalfe in DPPC, egg PC, DPPC/cholesterol, and egg PC/cholesterol vesicles,^2^ by Pope *et al.* in DMPC bilayers,^3^ and by Boden *et al.* in DMPC bilayers.^4^ (2) Hydrocarbon sites. Binding sites located in some unspecified way within the chain region of DPPC, egg PC, DPPC/cholesterol, and egg PC/cholesterol were suggested to exist by Colley and Metcalfe.^2^ In contrast to headgroup sites, hydrocarbon sites produce no shift of the resonance of the headgroup *N*-methyl protons. Partitioning of solutes into the hydrocarbon chain region is also predicted by the theory of Marqusee and Dill.^5^ López Cascales *et al.*(6) found that at surgical levels of benzyl alcohol concentration in DMPC bilayers (solute to lipid mole ratio *R*_*S*_ of ≈0.04), benzyl alcohol molecules were located in chain end region – *i.e.*, in a region populated by carbons in position *i* = 9 to *i* = 12 after being initially placed in the center of the bilayer. A site in which the hydroxyl group of benzyl alcohol interacts with the carbonyl group of the linkage to the hydrocarbon chain while the aromatic ring is placed along the first seven methylene segments of the chain was proposed to exist in potassium stearate/potassium oleate bilayers by Boden *et al.*(7)

**Figure 1:**
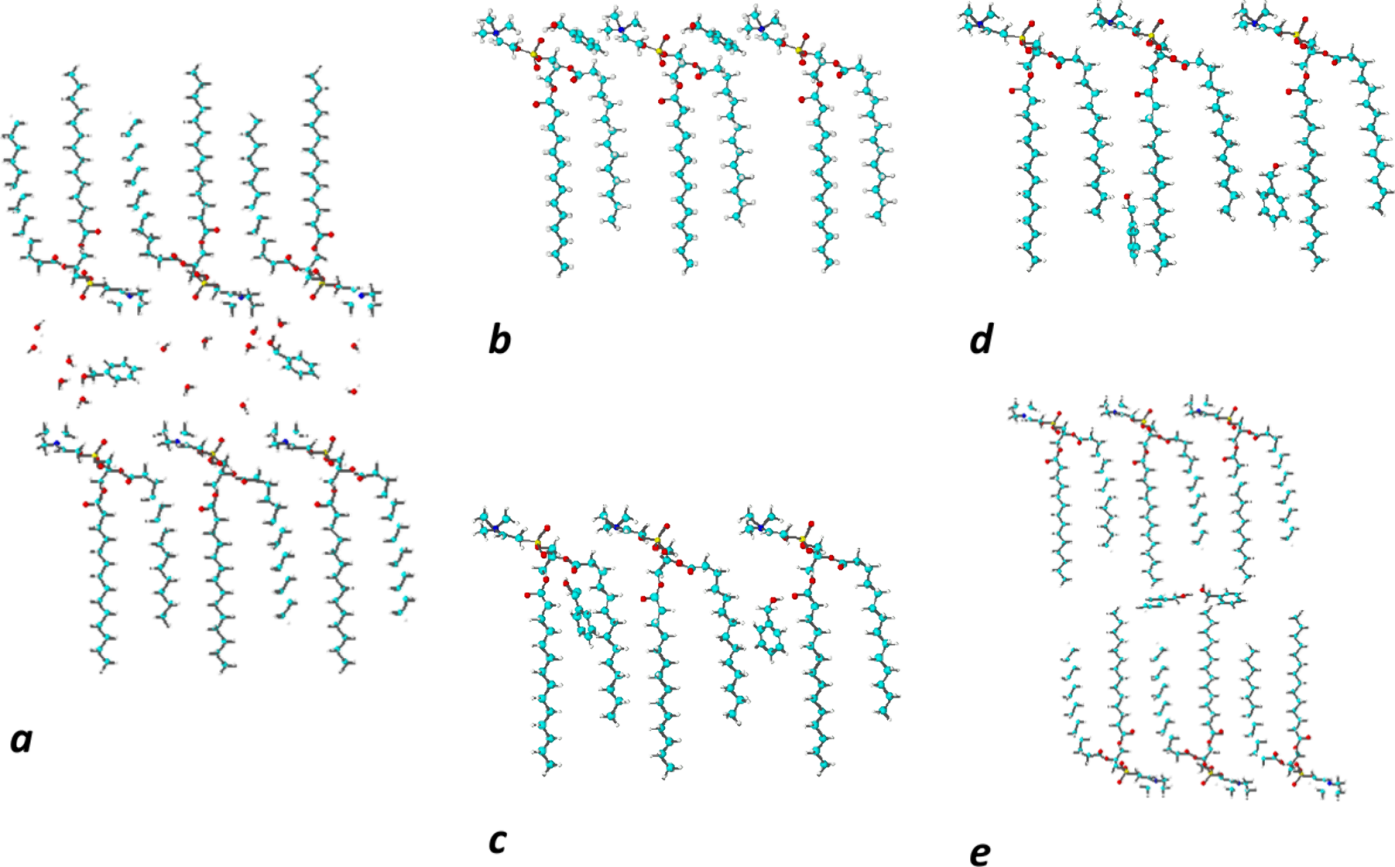
Binding sites of benzyl alcohol. (a) Site I – benzyl alcohol molecules are located in the interbilayer water layer. (b) Site II – benzyl alcohol is located in the polar headgroup region and its hydroxyl group might form hydrogen bond with the phosphate group of the DMPC molecule. (c) Site III – benzyl alcohol is inserted between the upper parts of neighboring hydrocarbon chains and its hydroxyl group might form hydrogen bond with the carbonyl group of the *sn*-1 (right molecule) or *sn*-2 (left molecule) lipid chain. (d) Site IV – benzyl alcohol molecules are intercalated between lower part of the chains in the hydrocarbon region. (e) Site V – benzyl alcohol forms a layer between chain ends of opposing lipid monolayers. Color code: white – hydrogen, light blue – carbon, dark blue – nitrogen, red – oxygen, yellow–phosphate. Adapted with permission from ref (53). Copyright 2000 University of Leeds and Šturcová.

In principle, benzyl alcohol could be located in any of the following binding sites (Figure 1): (1) Site I. This site represents those benzyl alcohol molecules that partition into the aqueous layer separating adjacent bilayers (Figure 1a). (2) Site II. Benzyl alcohol is localized in the polar headgroup region and could be hydrogen bound by its hydroxyl group to the phosphate group of the DMPC headgroup (−O–H···O–PO(OR)_2_) as has been suggested by Boden *et al.*,^8^Figure 1b. (3) Site III. In this site, the aromatic ring of benzyl alcohol is intercalated between the hydrocarbon chains of lipid along the first few segments of the chains and the hydroxyl group of benzyl alcohol molecule possibly interacts with the carbonyl group of *sn*-1 or *sn*-2 lipid chain via a hydrogen bond, Figure 1c. (4) Site IV. This is a site in which the benzyl alcohol molecule is intercalated between the neighboring lipid chains further down in the hydrocarbon bilayer region, Figure 1d. (5) Site V. Benzyl alcohol is accumulated in the center of the bilayer and forms a layer between chain ends of opposing lipid monolayers, Figure 1e. It is presumed that benzyl alcohol molecules can partition simultaneously between these binding sites. In each of these sites, benzyl alcohol will have a distinctive value of chemical potential. The value of the chemical potential will be a function of hydration, benzyl alcohol concentration, and temperature. The relative values of the chemical potential among the sites will determine the population distribution. Since the partition coefficient of benzyl alcohol between DMPC vesicles and water (*K*) is equal to 329^9^ (*K* is expressed as the ratio of the benzyl alcohol mole fraction in DMPC vesicles and in bulk solution), it is presumed that all benzyl alcohol is located within the bilayer, *i.e.*, that site I is not significantly populated.

The presence of benzyl alcohol in the bilayer will affect orientational order of the lipid chains. It is expected that the effect of benzyl alcohol on the order profile of the chains will be dependent on the location of the solute and that the effect of benzyl alcohol will be combined with the effect of hydration.

### Dependence of the Surface Density of Amphiphile Molecules on the Average Order Parameter of the Amphiphile Hydrocarbon Chain

#### Bilayer Models

Rotation about the carbon–carbon single bonds in lipid hydrocarbon chains is the molecular basis for the fluidity in the liquid crystalline phase of phospholipid bilayers. The properties of the fluid phase are a result of a weighted average over all interconverting rotational states of the chains and can be calculated by the methods of statistical mechanics.

In order to describe conformation of isolated chains, statistical mechanical methods exist allowing for the intramolecular conformational restrictions on the chain conformation, using the rotational isomeric approximation. Steric repulsions within the chain are considered, but chain–chain interactions are usually neglected.^10^ For lipid chains in bilayers, the chains are no longer isolated, intermolecular chain–chain interactions place steric restrictions on chain conformation. The most important interactions determining the equilibrium lateral chain packing density are the repulsive excluded-volume interactions and the attractive van der Waals interactions between the chains. In addition, the surface terms (hydrophobic, electrostatic, and hydration contributions) are present. According to the approach chosen to account for the interactions involved, various theoretical models of lipid bilayers exist: the mean-field molecular theory of Marčelja,^11^ the model of Nagle^12^ that is based on a triangular lattice, and also other two-dimensional lattice models. Numerical methods, molecular dynamics simulation and Monte Carlo calculations, were also used to reproduce the properties of real bilayers.^13^

#### Ordering State and Structural Characteristic of the Bilayer

The lateral packing density of hydrocarbon chains forming the bilayer is usually described in terms of either the average area per lipid molecule ⟨*A*⟩, the average lipid cross sectional area *a*_*L*_, or the hydrocarbon layer thickness *d*_*CH*_.^7,13−15^ In solute-free bilayer systems, these parameters can be determined by the application of low angle X-ray diffraction using the so-called Luzzati method.^16^ However, this method cannot be applied in those solute-containing bilayers, in which the distribution of the solute within the bilayer is unknown and if the Luzzati method is to be applied to such data, assumptions about the solute distribution must be made prior to calculations (see Results).

Deuterium NMR spectroscopy can be used to test theoretical predictions of bilayer models and to characterize the order of the hydrocarbon chains.^17^ The molecular interpretation of the deuterium order parameter can be accomplished at different levels of sophistication.^18,19^ Qualitatively, the amphiphile molecules can be visualized as being anchored by their polar groups at the lipid/water interface. This close packing of lipid headgroups imposes steric constraints on the hydrocarbon chains reducing their conformational and motional freedom. As a result, the order parameter in the upper part of the chains is roughly constant and this region is called the plateau region (for DMPC this is typically between the carbon positions *i*, from 3 to 9). The steric restrictions are relieved toward the ends of chains, which is reflected in decrease of the chain order (Figure S7 right, Supporting Information).

The average order parameter ⟨*S*⟩ is considered to be the measure of the average orientational order of the chains and for DMPC bilayers is defined as
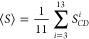
1where *S*_*CD*_^*i*^ is the C–D bond deuterium order parameter corresponding the CD_2_ segment in position *i* in the chain. The order parameter for position *i* = 3 is chosen as the start of the averaging since it was shown by Seelig and Seelig^20^ and Oldfield *et al.*(21) that the *sn*-2 chain is initially extended parallel to the bilayer surface, and, after the *i* = 2 position, it is perpendicular to this plane. The last (*i* = 14) CD_3_ group segment is not included in the calculation of the average order parameter since this segment is expected to be in that portion of the hydrocarbon part of the bilayer, where the chains from the two opposing layers are overlapping.

It is possible to assume that there is a specific functional form between the lateral chain packing density (reflected in the structural parameters ⟨*A*⟩, *d*_*CH*_, and *a*_*L*_ – see Supporting Information for definitions) and the chain organization (ordering). This assumption is supported by the work of Mely *et al.*(14) who have shown that an increase of the average surface lipid area ⟨*A*⟩ implies a less ordered bilayer since the expansion of the lattice leads to larger conformational freedom for the hydrocarbon chains. The authors also showed that two potassium laurate bilayer preparations of different temperatures, but with the same average area per molecule, had the same order parameter profiles. The X-ray diffraction studies of the DMPC/water systems show^22^ that when hydration is increased, the average area per chain *a*_*CH*_ (while *a*_*L*_ = 2 *a*_*CH*_) increases as well. This is a further support for the abovementioned relationship between the lateral chain packing density and the chain ordering since increased hydration of the lipid molecules implies reduced steric constraints for the hydrocarbon chains.

The order parameter is related to the average chain geometry and thus, it can provide information on the bilayer structure without having to deal with complex theories. Seelig and Seelig^20^ obtained information from the order parameter *S*_ρ_ on some aspects of the bilayer structure through geometric consideration of the chain structure. This model was further refined by Schindler and Seelig.^23^ Dill and Flory^24^ established the relationship between the molecular order parameter *S*_ρ_ and the normalized surface density σ (defined as the ratio of the area per phospholipid molecule in the crystal, *a*_*L*_^0^= 40.8 Å^2^, to the area per phospholipid, *a*_*L*_) through use of a lattice model of chain packing. Nagle^25^ obtained a relation between the C–D bond order parameter for the *n*th CD_2_ group within the plateau region *S*_*CD*_^*P*^ and the mean distance ⟨*d*⟩ that the chain travels along the bilayer normal at the same CD_2_ group.

An empirical correlation was shown to exist between the average deuterium order parameter ⟨*S*⟩ of the hydrocarbon chains and the inverse average area per chain (1/*a*_*CH*_) by Boden *et al.*(7) The correlation was based on deuterium NMR spectroscopy and X-ray diffraction studies in the *L*_α_ phase of soap/water and phospholipid/water bilayers. The functional form of the dependence of the ordering state of the chains on (1/*a*_*CH*_) was found to be linear. The correlation was argued to be universal, independent of the chemical structure of the amphiphilic molecule: its hydrophilic group, the number of chains per headgroup, and the chain length. The correlation was also claimed to retain linearity over the entire range of stable lamellar phases. The same approach as the one in Boden *et al.*(7) was utilized by Aggeli *et al.*(26) who studied the ordering state and structure of phospholipid (DMPC)/water systems over the whole liquid-crystalline phase of this lipid. A correlation between the average order parameter ⟨*S*⟩ and the inverse value of the average surface area per hydrocarbon chain (1/*a*_*CH*_) in DMPC/water bilayers was found and was used as a calibration for systems containing solutes dissolved in this phospholipid (DMPC) bilayer.

In order to learn about benzyl alcohol–lipid bilayer interactions, the phase behavior of DMPC/water/benzyl alcohol system has been looked at in this work.

Behavior of the parameters of mesoscopic structure of the DMPC/water bilayer and of the lipid molecule itself upon addition of benzyl alcohol solute has also been studied using low angle X-ray diffraction and NMR spectroscopy.

In order to enable quantifications of the structural parameters, an empirical mathematical bilayer model has been developed that is most likely representing the relationship between intrinsic bilayer forces and the bilayer elasticity.

The existence of several binding sites for benzyl alcohol has been verified and the population distribution between these sites has been determined. The effect of benzyl alcohol on the chain order profiles has been assessed and the impact of hydration on interaction of benzyl alcohol with DMPC bilayer has been evaluated.

## Materials and Methods

### Materials and Sample Preparation

1,2-Dimyristoyl-*sn*-glycero-3-phosphocholine (DMPC) was obtained from Lipid Products (The Granary Brewer Street, Bletchingley, Redhill, Surrey, RH1 4QP, United Kingdom) in the form of a chloroform/methanol solution. The purity of a batch of DMPC was checked by thin layer chromatography when the lipid was in solution form and as an eluant chloroform/methanol/water solution (65:35:3, v:v) was used. DMPC with the perdeuterated *sn*-2 chain (d_27_DMPC) was synthesized according to Gupta *et al.*(27) and purified by Lipid Products (United Kingdom). The stability of perdeuterated DMPC was checked by differential scanning calorimetry (DSC). High purity benzyl alcohol (99+%, gold label) was obtained from Aldrich Chemical (now: Sigma-Aldrich, St. Louis, Missouri, United States), and deuterium-depleted water was also purchased from Aldrich Chemical (USA). All of these compounds were used without further purification.

DMPC was transferred in solution form into glass sample tubes and the excess solvent was evaporated under a stream of dry nitrogen. The remaining solvent and water were removed by pumping on a vacuum line for approximately 48 h. The dry mass of DMPC was determined gravimetrically using an electronic balance. Typical dry mass for a single sample was approximately 20–30 mg of the lipid. In order to add the required quantities of water and benzyl alcohol, microsyringes were used and the masses of these components were also determined gravimetrically. For ^2^H NMR experiments, deuterium-depleted water was used for hydration. After additions, the sample tubes were sealed with parafilm and centrifuged to ensure no water nor benzyl alcohol was present at the top of the tubes and these components were not lost during flame sealing. After centrifugation, the parafilm was removed and the sample tubes flame-sealed. Once sealed, the content of the tubes was thoroughly mixed by centrifugation and samples were left to equilibrate at approximately 313 K in water bath for 7 days with occasional centrifugation. After equilibration, the sample tubes were opened and a rubber plug wrapped in PTFE tape was inserted into the tubes just above the sample content to avoid demixing during an NMR experiment. After the plug insertion, the sample tubes were flame-sealed again. For an X-ray diffraction experiment, a fraction of the content of the sample tubes was transferred into an X-ray capillary using drawn glass pipet, the capillary was then centrifuged and flame-sealed. Between experiments, the samples were stored at approximately 277 K to prevent degradation.

### NMR Spectral Acquisition

^31^P and ^2^H NMR spectra were recorded on a Bruker MSL 300 spectrometer at a magnetic field of 7 T operating in the quadrature detection mode. For ^2^H NMR, a high-power broadband probe with saddle coil of 10 mm diameter was used and tuned to 46.073 MHz. The ^2^H NMR experiments were performed by the use of solid echo pulse sequence 90°−τ–90°−τ–FID.^28^ The ^2^H NMR spectra were acquired with a 90° pulse of 8–11 μs pulse length, pulse separation of 18 μs, recycle delay of 500 ms, and sweep width of 250 kHz. The spectra typically had 4096 data points, between 10,000 and 100,000 scans. The time domain was reduced, the size was increased, and left shift and line broadening of 0–40 Hz were applied to the free induction decays before Fourier transformation in order to reduce noise, to obtain good digitization and to locate echo maximum.

For ^31^P NMR, a high-resolution variable spin probe was used and tuned to 121.497 MHz. The proton-decoupled ^31^P NMR spectra were obtained in inverse-gated heteronuclear decoupling NMR experiments. The pulse length was typically 5 μs, the recycle delay was 2 s, and a sweep width of 50 kHz was used. FIDs of 1,000 to 35,000 scans were acquired, they consisted of 4 096 data points, and reduction of time domain and line broadening of 0 to 50 Hz was applied to them before Fourier transformation into spectra. The sample temperature was controlled to ±1.0 K with the spectrometer temperature control unit.

An assignment of the individual peaks in Pake powder ^2^H NMR spectra to the corresponding methylene groups was obtained by comparison with published results for specifically deuterated phospholipids^21^ and by assuming that the segmental order parameter *S*_*CD*_^*i*^ decreases monotonically toward the end of the hydrocarbon chain. *S*_*CD*_^*i*^ values were calculated according to eq 2
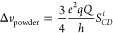
2assuming negative sign, a value of 170 kHz was used^7^ for the quadrupolar coupling constant .

The two principal values, σ_⊥_ and σ_//_, of the chemical shielding tensor of axial symmetry were determined from ^31^P NMR spectra in the following way: σ_⊥_ was read at the 84–88% of the peak height of the high intensity shoulder of the spectrum,^29^ and σ_//_ was determined from the half-height of the low intensity shoulder.

### Determination of Bilayer Dimensions Based on X-ray Diffraction Data

Lamellar repeat distance *D* was determined by X-ray diffraction. The X-ray diffraction experiments were performed according to Turnbull^22^ using a home-built pinhole camera operating with a copper Kα source at a wavelength λ of 1.54 Å. Fogging of the photographic plate by an undiffracted X–ray beam was prevented by the use of a beam stop. Temperature control was attained by the use of a heating block and copper/constantan thermocouple arrangement and had a temperature stability of ±0.5 K. Samples in X-ray capillaries were left to allow temperature equilibration before exposure. A typical exposure time of 3 h was required to observe the low-angle diffraction pattern associated with the one-dimensional lamellar lattice, and the diffraction ring diameters were determined by use of a Hilger and Watts microphotometer connected to an analogue-to-digital converter unit. The densitometer traces were recorded by a computer. The following parameters of the mesoscopic bilayer structure were calculated from the lamellar repeat distance *D*: average surface area per lipid at the bilayer/water interface denoted as ⟨*A*⟩, the maximum and the minimum hydrocarbon layer thickness *d*_*CH*_^*max*^ and *d*_*CH*_^*min*^, and the minimum as well as the maximum thickness of the interbilayer water layer *d*_*W*_^*min*^ and *d*_*W*_^*max*^. The accuracy of the lamellar repeat distance *D* was initially determined only for the high hydration of *R*_*W*_ = 23 (see Table S3 in Supporting Information), but not for the low hydration of *R*_*W*_ = 8 (see Table S4 in Supporting Information). Later, it was not possible to re-examine the relevant X-ray diffraction images since they were no longer available to the author of the current work – as a consequence, the accuracy of the lamellar repeat distance *D* and of the associated structural parameters stated in the relevant tables and the error bars displayed in the relevant graphs are stated or displayed only for the higher value of the limited hydration used, *i.e.*, for hydration *R*_*W*_ = 23.

## Results

The fluid lamellar phase (*L*_α_) may be considered to be a smectic A mesophase since the hydrocarbon chains are in the fluid (disordered) state.^16^ The interaction of benzyl alcohol with DMPC bilayers was studied in their fluid lamellar phase in this work.

The fluidity of the bilayer in *L*_α_ phase means that curvature can occur that leads to formation of liposomes. Such a change in meso- and macrostructure from planar to spherical geometry is assumed to approximately correlate with the swelling limit of the phase *R*_*W*_^*SL*^ and it is assumed here that such a change does not take place below *R*_*W*_^*SL*^ even though not all the water molecules that are added to the lipid can be expected to go perfectly between the stacks of bilayers^30^ and thus defects may be present in the form of regions with excess water phase even before the two phase region is entered thermodynamically (great care was taken during sample preparation to make the systems as homogeneous as possible).

Several macrostructural “transition points” can be defined in the *L*_α_ phase of DMPC and they can be identified either by the variation of the lamellar repeat distance (*D*) or the optical density (turbidity) as a function of hydration *R*_*W*_. The saturation point (*R*_*W*_^*SP*^) was suggested by Zhang^31^ to correspond to the hydration required to form a primary hydration shell around the phospholipid headgroup. For *R*_*W*_ ≤ *R*_*W*_^*SP*^, *D* is assumed to be approximately independent of hydration because any increase in the interbilayer water layer thickness *d*_*W*_ is compensated for by a corresponding decrease in the bilayer thickness *d*_*t*_ due to rapid expansion of the bilayer caused by hydration of the headgroups. For *R*_*W*_ > *R*_*W*_^*SP*^, *D* increases approximately linearly with hydration until the curvature point (*R*_*W*_^*CP*^) is reached. This transition point is believed to be associated with a change in phase structure from planar to curved bilayers – such a structural change is accompanied by an increase in turbidity as well. The turbidity continues to increase with hydration until a liposomal dispersion is formed at the dispersion point (*R*_*W*_^*DP*^); thereafter, it remains constant. The swelling limit, *R*_*W*_^*SL*^, is believed to occur before the dispersion point, *R*_*W*_^*DP*^, and hence the liposomes are suggested to be polyhedral rather than spherical at *R*_*W*_^*SL*^. Due to the probability that liposomes have water cores, it is believed that not all of the water in the *L*_α_ phase is located between the bilayers, and hence a more gradual increase in *D* with hydration is observed.

The lipid bilayer models that were developed and the interaction of benzyl alcohol with DMPC bilayers that was studied in this work were all conducted in fluid lamellar *L*_α_ at hydration levels at or above the saturation point *R*_*W*_^*SP*^, but well below the curvature point *R*_*W*_^*CP*^, so that the water added to the lipid could be assumed to be localized only between the lamellae, *i.e.*, it was possible to assume that no excess water was present in the sample while the lipid lamellae remained planar and their stoichiometry was known. For DMPC bilayers, this meant working at hydration *R*_*W*_ between approximately 8 to approximately 25 water molecules per one lipid molecule. Thus, the samples used in this study may be visualized as ensembles of domains – each domain consisting of a stack of parallel bilayer aggregates and the domain normal axes oriented randomly with respect to chosen external reference axis – this is a similar arrangement to the arrangement of crystallites in a polycrystalline powder sample. The levels of hydration corresponding to the individual macrostructural transition points described above are presented in Table S1 (Supporting Information). Comparison with literature values^30^ of the values of structural parameters (the average cross-sectional area of the hydrocarbon chain *a*_*CH*_, Table S2 in Supporting Information) obtained from X-ray diffraction data under the above-mentioned assumptions about the bilayer planarity shows that the analysis presented in this work provided realistic estimates of bilayer structural parameters. Such analysis was chosen for this work also due to the fact that it enabled us to use the samples of the same preparation and the same geometry as those samples that were used for investigation by NMR spectroscopy (see below).

### Dependence of Surface Density of Amphiphile Molecules on the Average Order Parameter of the Amphiphile Hydrocarbon Chain

#### Model of the Dependence of Surface Density of Amphiphilic Molecules on the Order Parameter

##### (a) Empirical Mathematical Model

**Figure 2:**
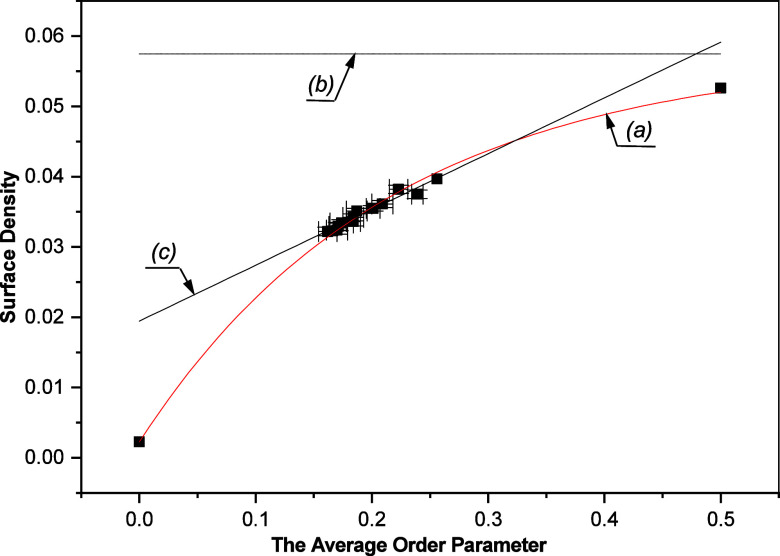
Surface density (1/*a_CH_*) dependence on the average order parameter ⟨*S*⟩ based on DMPC/water bilayer data and two hypothetical data points representing limiting behavior of lipid bilayer. The curves represent the following: (a) The exponential fit eq 7 to the data with 1/*a_CH_^e^*= 0.057 Å^–2^, *K*_*S*_ = 0.216, and 1/*a_CH_^max^*= 0.002 Å^–2^. (b) The asymptote 1/*a_CH_* = 1/*a_CH_^e^* of the exponential fit in (a). (c) The linear dependence with *p*_1_ = (0.019 ± 0.001) Å^–2^ and *p*_2_ = (0.079 ± 0.006) Å^–2^ determined from a fit to the DMPC data. Reprinted with permission from ref (53). Copyright 2000 University of Leeds and Šturcová.

Using the data obtained by X-ray diffraction (*a*_*CH*_) and by ^2^H NMR (the order parameters) on DMPC/water bilayers at a temperature of 313 K (Table S2, Supporting Information), it is possible to show that the surface density of chains (1/*a*_*CH*_) is linearly dependent on the average order parameter ⟨*S*⟩ within the experimentally determined range of *a*_*CH*_ and ⟨*S*⟩ values (Figure 2).

**Scheme 1:**
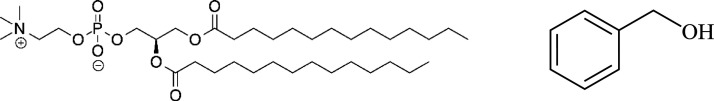
Structure of 1,2-Dimyristoyl-*sn-*glycero-3-phosphatidylcholine (DMPC; Left) and Benzyl Alcohol (Right)

In order to obtain information about behavior of surface density (1/*a*_*CH*_) outside the experimentally determined range of *a*_*CH*_ and ⟨*S*⟩ values, it is useful to consider the following two limits: (1) a limit corresponding to the most ordered state of the bilayer, *i.e.*, a bilayer in the crystalline state, when |⟨*S*⟩| = 0.5 and the average cross-sectional area of chain is at its minimum *a*_*CH*_^*e*,*r*^ = 19 Å^2^,^32^ while the surface density is at its maximum, (1/*a*_*CH*_^*e*,*r*^) = 0.053 Å^–2^; (2) a limit corresponding to the most disordered bilayer, in which case we assume that |⟨*S*⟩| = 0 and the average cross-sectional area of chain is at its maximum, *a*_*CH*_^*max*^ = 442.92 Å^2^, the corresponding minimum surface density is 1/*a*_*CH*_^*max*^ = 0.002 Å^–2^. The value of the maximum average cross-sectional area of chain has been obtained assuming *a*_*L*_^*max*^ = 2*a*_*CH*_^*max*^, where *a*_*L*_^*max*^ is the maximum average cross-sectional area of lipid. The value of *a*_*L*_^*max*^ was estimated from the radius of gyration of the lipid molecule *R*_*g*_: the radius of gyration was taken to be 80% of the distance between the C_3_ atom of the glycerol backbone and C_14_ atom of the *sn*-1 chain in the DMPC molecule (Scheme 1), which was estimated from molecular model to be 20.99 Å. As can be seen from Figure 2, values of (1/*a*_*CH*_) and ⟨*S*⟩ obtained by measurement and the hypothetical data points representing the two limiting cases described above indicate that surface density (1/*a*_*CH*_) varies nonlinearly with ⟨*S*⟩. In the following treatment, the functional form of this nonlinear dependence is obtained.

It follows from the works of Seelig and Seelig,^20^ Schindler and Seelig,^23^ Dill and Flory,^24^ De Young and Dill,^15^ Boden *et al.*,^7^ and Aggeli *et al.*(26) that the properties related to surface density (1/*a*_*L*_) or (1/*a*_*CH*_) vary with the order parameter. These works are the basis of a mathematical model, which describes the relationship between the surface density and the order of the amphiphilic molecules in the bilayer. Further, we will also utilize analogy with mathematical models describing phenomena related to density or concentration, *e.g.*: (a) the mathematical model of saturation of water with oxygen, in which it is assumed that the change of the concentration of oxygen in water with time is proportional to the difference of the maximum concentration of oxygen and the actual concentration:^33^
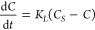
3where *K*_*L*_ is the oxygen permeability coefficient, *C*_*S*_ is the maximum concentration of oxygen in water, and *C* is the actual concentration of oxygen in water at time *t*; (b) charging of a capacitor can be described by the same equation^34^ saying that the change of the surface density of charge with time is proportional to the difference of the maximum surface density and the actual density:
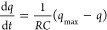
4where *q*_*max*_ is the maximum surface density of charge, *q* is the actual surface density at time *t*, *R* is the resistance, and *C* is the capacity. It follows from eqs 3 and 4 that the mathematical model of oxygen saturation of water as well as the model of capacitor charging contains an important condition of the process of saturation or charging – the concentration of oxygen in water and the surface density of charge cannot increase to any arbitrary value, but they are restricted by the maximum concentration of oxygen (at given temperature) and by the maximum surface density of charge.

We will look for a mathematical model of the dependence of surface density of lipid molecules on the average order parameter under these assumptions: (i) the surface density increases with modulus of the average order parameter; (ii) the surface density of the amphiphilic molecules is not allowed to increase to an arbitrary value, but it is restricted by the surface density of the molecules in the bilayer in crystalline state (1/*a*_*L*_^*e*^), which is considered to be the maximum surface density; (iii) we assume that the surface density approaches the minimum surface density (1/*a*_*L*_^*max*^) as the modulus of the average order parameter approaches zero. Based on these three assumptions and utilizing the analogy with eqs 3 and 4, we can define a mathematical description of the dependence of surface density on the average order parameter: the change of surface density *d*(1/*a*_*L*_) is proportional to the change of the modulus of the average order parameter d|⟨*S*⟩| and to the difference of the maximum surface density and the given density  corresponding to the given value of |⟨*S*⟩|:
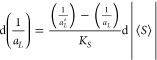
5where *K*_*S*_ is the coefficient of proportionality. Under the assumption that surface density is (1/*a*_*L*_^*max*^) for ⟨*S*⟩ = 0 (*i.e*., requirement (iii) above is valid), the following function, which expresses the dependence of surface density (1/*a*_*L*_) on the average order parameter ⟨*S*⟩ is the solution of the eq 5:
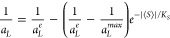
6

Considering that the area per molecule is twice the cross-sectional area of hydrocarbon chain, *a*_*L*_ = 2*a*_*CH*_, the surface density of the hydrocarbon chains depends on the average order parameter:
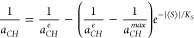
7

It means, that with increasing modulus of the order parameter, *i.e*., with increasing order of the molecules, the surface density of the hydrocarbon chains increases until it reaches the maximum value of (1/*a*_*CH*_^*e*^).

By expansion of the exponential function, eq 7 into Taylor series and by considering the first two terms of this expansion, one can obtain linear dependence of the surface density on the average order parameter. The offset *p*_*1*_ and the slope *p*_*2*_ of the linear dependence can be expressed in terms of relationships to the coefficient of proportionality *K*_*S*_, to the maximum surface density of the hydrocarbon chains in the bilayer in crystalline state (1/*a*_*CH*_^*e*^), and to the minimum surface density (1/*a*_*L*_^*max*^). The linear dependence is a tangent to the exponential curve, eq 7 at the point |⟨*S*_0_⟩| with offset *p*_*1*_ and slope *p*_*2*_.

##### (b) Analysis of the Experimental Data in Terms of the Mathematical Model

The data obtained on DMPC/water bilayer systems at temperature 313 K (Table S2, Supporting Information) and the two hypothetical data points corresponding to the limiting values (1/*a*_*CH*_^*e*,*r*^) and (1/*a*_*CH*_^*max*^) have been fitted to the function describing the surface density (1/*a*_*CH*_) dependence on the average order parameter ⟨*S*⟩ given by eq 7. It has been found by fitting that 1/*a*_*CH*_^*e*^ = (0.057 ± 0.001) Å^–2^, *K*_*S*_ = (0.216 ± 0.010), while 1/*a*_*CH*_^*max*^ was kept constant at 0.002 Å^–2^, the correlation coefficient was *R* = 0.998. During the fitting procedure, no weighting has been used. The graph of this function is in Figure 2 (line *a*).

The DMPC/water bilayer data at 313 K were, apart from the exponential function, eq 7, fitted also to a linear function, the parameters of which can also be determined from the Taylor expansion of the exponential function eq 7. The parameters of the linear dependence, *p*_1_ and *p*_2_, found by fitting of the DMPC/water data (Table S2) are presented in Table 1 (system (b)) and the graph of the dependence is in Figure 2 (line c). During the fitting procedure, no weighting has been used. As can be seen from Table 1, the values of parameters *p*_1_ and *p*_2_ obtained in this work are close to the ones found for the same system presented in Aggeli *et al*.^26^ (Table 1, system (a)).

**Table 1:** Parameters of Linear Dependence of the Surface Density (1/*a_CH_*) on the Average Order Parameter ⟨*S*⟩^c^

system	*p*1 [Å^–2^]	*p*2 [Å^–2^]	χ_*R*_^2^	R
DMPC/water^a^	0.023 ± 0.001	0.067 ± 0.006		
DMPC/water^b^	0.019 ± 0.001	0.079 ± 0.006	0.54	0.975

a Aggeli *et al*.^26^

b Parameters found in this work by fitting DMPC/water data from Table S2.

c Reprinted with permission from ref (53). Copyright 2000 University of Leeds and Šturcová.

Since the DMPC/water bilayer data are data with errors in both coordinates, the following formula has been used to calculate the value of chi-squared:^35^
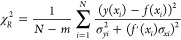
8where *N* is the number of data points used, *m* is the number of fitting parameters varied during the fitting procedure, *y*(*x*_*i*_) is the measured surface density of chains, *f*(*x*_*i*_) is the surface density of chains calculated from the function fitted to the data, σ_*xi*_ and σ_*yi*_ are, respectively, the *x* and *y* standard deviations for the −th point, and *f’*(*x*_*i*_) is the first derivative of the fitting function.

When only the DMPC/water bilayer data were used for calculation of χ_*R*_^2^, *i.e.*, when using the data for the average order parameter values from the interval ⟨0.162; 0.223⟩ (temperature of 313 K, Table S2), the values for both the exponential fit (χ_*R*_^2^ = 0.85) and for the linear fit (χ_*R*_^2^ = 0.54) were smaller than 1.0, which indicated that the errors had probably been overestimated. According to the chi-squared value, the linear fit described the data better than the exponential fit. This was caused by the fact that the exponential fit used the two hypothetical data points apart from DMPC/water bilayer data at 313 K and therefore represented a wider range of order parameter values. The chi-squared value for the exponential fit was χ_*R*_^2^ = 0.88, when the two hypothetical data points were included. Clearly from Figure 2, the exponential fit was a better model when the entire range of order parameter values was considered.

We attempted to expand the range of both the area *a*_*CH*_ and the average order parameter ⟨*S*⟩ values for DMPC bilayers by using samples with high water content and at high temperature. However, the hydrations available were restricted by the requirement to preserve planar geometry of bilayers, which for DMPC means to work at hydrations lower than ≈25 water molecules per lipid molecule. Further, the temperature had to be kept below 60 °C to ensure stability of the lipid over the duration of the experiment and also to work within the range of temperature values for which the density of liquid hydrocarbon in bilayer was known.^36^ Thus, the range for DMPC bilayers was not expanded significantly – the lowest attained value for the average order parameter was −0.144 and the largest area was 32.3 Å^2^. DMPC molecule disorders more and attains larger area (spanning the whole range of lamellar phases) while preserving planar geometry of the bilayer under the influence of some solutes.

The exponential dependence of the surface density (the inverse chain area) (1/*a*_*CH*_) on the average order parameter ⟨*S*⟩ described well the behavior on the whole range of the stable lamellar *L*_α_ phase, for which the values of *a*_*CH*_ vary from ≈25 Å^2^ to ≈40 Å^2^. If necessary, the exponential dependence can be replaced by a linear relationship on the individual subintervals of the average order parameter values corresponding to the lamellar *L*_α_ phase. The exponential dependence did not predict phase transitions and it can be expected that different dependences will describe relationship between the average order parameter and the surface density for the phases other than fluid lamellar *L*_α_.

### Phase Behavior of DMPC Bilayer Systems with Added Benzyl Alcohol

Turnbull^22^ used DSC over a wide range of hydration *R*_*W*_ and solubilization (solute-to-lipid mole ratio) *R*_*S*_ to describe the effect of benzyl alcohol on lyotropic phase behavior of DMPC/water bilayers and proposed phase diagrams at various *R*_*W*_. Further, Turnbull^22^ employed low-angle X-ray diffraction and turbidity measurements to study the macro- and mesostructure of the DMPC/water/benzyl alcohol ternary system for solute concentration up to *R*_*S*_ of 1.5 and 1.2, respectively, and for a range of hydrations – all at a temperature of 313.15 K. As can be seen from Table S1 (Supporting Information), the values of the saturation point (*R*_*W*_^*SP*^), the curvature point (*R*_*W*_^*CP*^), the swelling limit (*R*_*W*_^*SL*^), and the dispersion point (*R*_*W*_^*DP*^) are seen to increase with increasing amount of benzyl alcohol dissolved in the DMPC bilayer, *i.e.*, benzyl alcohol loosens and fluidizes the bilayers and enables incorporation of greater amount of water.

In this work, phosphorus (^31^P) NMR spectroscopy was employed to determine the maximum concentration, *i.e.*, the solubility limit, *R*_*S*_^*SL*^, of benzyl alcohol that can be accommodated by the fluid *L*_α_ phase of DMPC without phase separation of excess benzyl alcohol – it means that the boundary between *L*_α_ and (*L*_α_ + BZA) phase was established. Precise determination of solute solubility limit *R*_*S*_^*SL*^ is necessary for correct application of X-ray diffraction for quantification of structural parameters (see below). This means that X-ray data have to be obtained on samples below the solute solubility limit to ensure lamellarity of the phase present. It is also necessary to ensure that the stoichiometric composition of the lipid bilayer is the same as the stoichiometric composition of the sample overall – such an assumption is valid in planar bilayers^16^ (Supporting Information).

**Figure 3:**
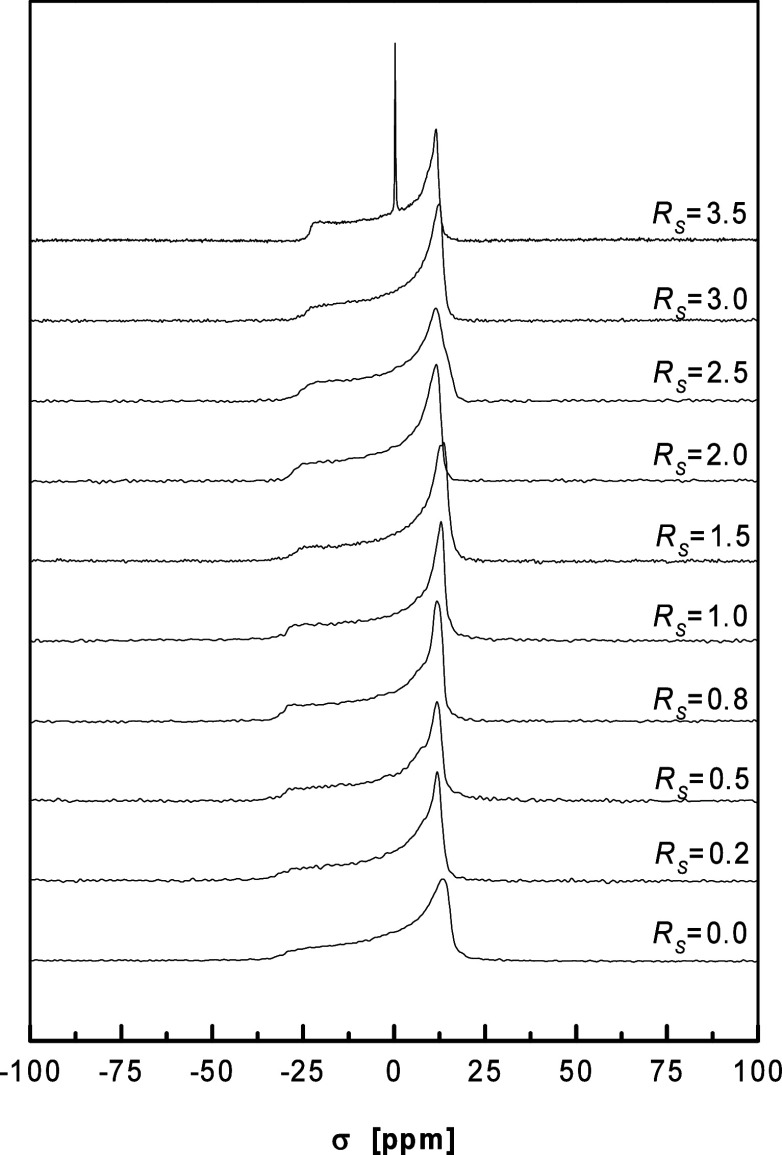
Proton-decoupled ^31^P NMR spectra of DMPC bilayers both in the presence and in the absence of benzyl alcohol at a hydration *R_W_* of 20 and temperature of 313 K. Reprinted with permission from ref (53). Copyright 2000 University of Leeds and Šturcová.

^31^P NMR spectra acquired on DMPC bilayers at a hydration *R*_*W*_ of 20 and temperature of 313 K at various benzyl alcohol concentrations are displayed in Figure 3. Under all conditions, the spectra represented an unoriented sample with a uniaxial chemical shielding tensor with negative anisotropy Δσ and were consistent with the existence of a lamellar bilayer phase, in which the lipid headgroups were undergoing rapid rotational motion around the bilayer normal.^37^ At the highest solute-to-lipid mole ratio, an isotropic line was present in the spectrum (see spectrum at *R*_*S*_ = 3.5 in Figure 3). The isotropic line was positioned at two-thirds of the shielding anisotropy Δσ and corresponds to those lipid molecules that partitioned into an excess benzyl alcohol phase either as small, fast tumbling aggregates or in a monomer form. The highest solute-to-lipid mole ratio *R*_*S*_, at which the spectra were devoid of the isotropic line, was identified as the solubility limit *R*_*S*_^*SL*^ – *i.e.,* as the maximum amount of benzyl alcohol that can be dissolved in the bilayer. At *R*_*W*_ = 20, the solubility limit was determined to be 3.0 benzyl alcohol molecules per one lipid molecule – a value consistent with *R*_*S*_^*SL*^ = 3.0 at *R*_*W*_ of 23 determined by X-ray diffraction.^22^ Further, hydration and temperature dependence of the solubility limit was also established – see Figures S4 and S5 in the Supporting Information.

### Structural Parameters of DMPC Bilayer in the Limited Hydration Regime as Determined by X-ray Diffraction

At a temperature of 313.15 K, Turnbull^22^ used X-ray diffraction to determine variation of lamellar repeat distance *D* with solubilization up to *R*_*S*_ = 4.0 for a high hydration *R*_*W*_ of 23 (Table S3, Supporting Information), which is the hydration level just below that of curvature point *R*_*W*_^*CP*^ = 24.5 ± 1.8 or *R*_*W*_^*CP*^ = 25.0 ± 2.5 in a solute-free bilayer system (Table S1, Supporting Information). The same was determined for a low hydration *R*_*W*_ of 8 at 313 K and solute concentration range 0.0 ≤ *R*_*S*_ ≤ 2.5 (Table S4, Supporting Information), while it is reminded here that a hydration level of 8 water molecules per lipid molecule is close to the saturation point *R*_*W*_^*SP*^ in solute-free bilayers (Table S1, Supporting Information). The two studied intervals of benzyl alcohol concentration, 0.0 ≤ *R*_*S*_ ≤ 4.0 and 0.0 ≤ *R*_*S*_ ≤ 2.5, at the two hydrations include the solubility limit of benzyl alcohol in DMPC/water bilayers, *i.e.*, *R*_*S*_^*SL*^ = 3.0 and *R*_*S*_^*SL*^ = 2.5 at the high (23) and the low (8) hydration, respectively.

**Figure 4:**
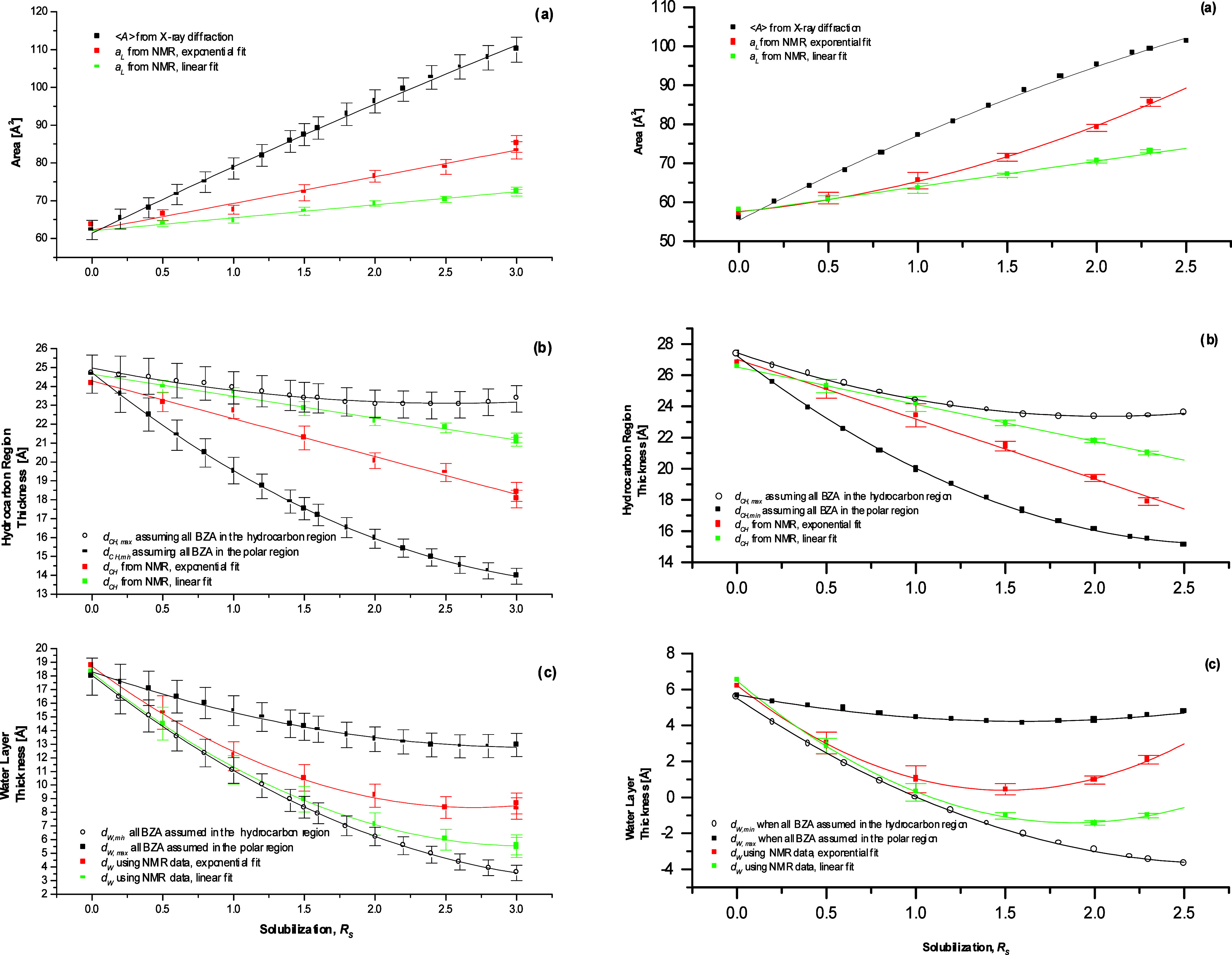
Parameters of the mesoscopic structure of DMPC bilayers at benzyl alcohol concentration *R_S_*, temperatures of 313 K for NMR and 313.15 K for X-ray. Left panels: hydration *R_W_* = 23 (X-ray diffraction data and NMR for solute-free bilayers) or *R_W_* = 24 (NMR of bilayers with benzyl alcohol). Right panels: hydration *R_W_* = 8. (a) Average surface area per lipid ⟨*A*⟩ (from X-ray) and average lipid cross-sectional area *a_L_* (from NMR); (b) the thickness of the hydrocarbon region of the bilayer (*d_CH_* – NMR; *d_CH_^max^* and *d*_*CH*_^*min*^ – X-ray); (c) the interbilayer water layer thickness *d_W_* (NMR and X-ray measurements) when all the solute is assumed to be in the polar headgroup region – site II (*d_W_^max^*) – and in the hydrocarbon region – sites III, IV, and V (*d_W_^min^*). See the text for calculation of the parameters. Values of the lamellar repeat distance *D* used for calculation of structural parameters (Tables S3 and S4, Supporting Information) were taken from Turnbull.^22^ Adapted with permission from ref (53). Copyright 2000 University of Leeds and Šturcová.

The values of lamellar repeat distance *D* determined by X-ray diffraction (Tables S3 and S4, Supporting Information) were used to evaluate the variation with solubilization of average surface area per lipid molecule ⟨*A*⟩ by the use of eq (S2) in the Supporting Information. Results of such calculations are displayed in Figure 4a (black lines and symbols). The assumption about bilayer planarity is sufficiently justified since Turnbull^22^ showed that at *R*_*S*_ = 1.2, the curvature point was as high as *R*_*W*_^*CP*^ = 46.1 ± 4.0 (Table S2, Supporting Information).

Variation with solute concentration of structural parameters such as hydrocarbon region thickness and the thickness of the interbilayer water layer was also examined. Since the solute distribution within the lipid bilayer was unknown prior to calculations, two extreme situations were considered: (*i*) All of the solute molecules were assumed to be localized within the polar headgroup region, whereby the headgroup region is assumed to consist of the choline group, the phosphate group, and the glycerol backbone, *i.e.*, benzyl alcohol is located in site II (Figure 1b and Figure S6i). (*ii*) All of the solute molecules were assumed to be placed in the nonpolar hydrocarbon portion of the bilayer, *i.e.*, in sites III, IV, and V, Figure 1c–e and Figure S6ii. In the first of these two extreme cases, the hydrocarbon region thickness was calculated according to eq (S12) (Supporting Information), and in the second case, eq (S13) (Supporting Information) was appropriate. The thus-obtained thickness values represent the minimum (*d*_*CH*_^*min*^) – case *i* – and the maximum (*d*_*CH*_^*max*^) – case *ii* – estimates of the hydrocarbon layer thickness (Figure 4b, black lines and symbols).

The minimum and the maximum bilayer thickness estimates (*d*_*t*_^*min*^ and *d*_*t*_^*max*^) can be found using eq (S7) (Supporting Information). In order to use eq (S7), the number of water molecules in the polar region per one lipid molecule *R*_*W*_^*P*^ was calculated first using eq (S8) (Supporting Information). When it is assumed that all benzyl alcohol molecules are bound in the headgroup region (site II), *R*_*W*_^*P*^ attains minimum values and the bilayer has minimum thickness *d*_*t*_^*min*^. In this case, eq (S11) (Supporting Information) is used to calculate the volume per lipid molecule, in which water can reside (Δ*v*_*P*_). The maximum thickness of the bilayer *d*_*t*_^*max*^ is obtained if all solute molecules are assumed to be located in sites III, IV, and V, in which case, eq (S10) (Supporting Information) is used for calculation of Δ*v*_*P*_ and *R*_*W*_^*P*^ attains maximum values – see also eq (S8) in the Supporting Information. Once the minimum (maximum) bilayer thickness, *d*_*t*_^*min*^ (*d*_*t*_^*max*^), is determined, the maximum, case *i*, (minimum, case *ii*) interbilayer water layer, *d*_*W*_^*max*^ (*d*_*W*_^*min*^), can be calculated according to eq (S15) in the Supporting Information – the results of these calculations are in Figure S6 and Figure 4c.

In these calculations, the partial molar volumes (*V*_*i*_) of water, benzyl alcohol, and DMPC were assumed to be equal to the molar volumes of the given compound and were taken to be 18.0, 103.5, and 669.2 cm^3^, respectively. The molar volume of water was calculated using the known bulk values of the molar mass and density, even though these bulk values most likely differ from those of water hydrating the polar lipid headgroups and of water localized between the lipid lamellae – the bulk values were seen as the best current approximation for water interacting with lipid headgroups. The molar volume of benzyl alcohol was determined from the values of the molar mass and density stated in the Aldrich Catalogue and was also an approximation since in each of the binding sites described above and in Figure 1, the value of the molecular volume may be different and is not known currently. Such uncertainty in molecular volume of a benzyl alcohol solute in lipid bilayer can be expected when the behavior of another solute – cholesterol – is considered: The volume of cholesterol in lipid bilayers above the chain melting temperature^40^ differed from its molecular volume when in the cholesterol crystal. In addition, the partial molecular volume of cholesterol was dependent on the lipid bilayer phase.^41^ The volume of the hydrocarbon portion of a DMPC molecule (*v*_*HC*_) was assumed to be 767.2 Å^3^ – the molar volume was determined to be 462.0 cm^3^ from specific volume measurements of Nagle and Wilkinson.^36^ The thickness of the polar region of the bilayer in the calculations was assumed to be identical with the length of the polar region of phosphocholine molecule perpendicular to the bilayer surface (*d*_*P*_) and was estimated to be ≈7.6 Å (Supporting Information).

#### Behavior of Structural Parameters at High Hydration

The average surface area per lipid molecule ⟨*A*⟩ was seen to increase with increasing benzyl alcohol concentration at the high hydration *R*_*W*_ of 23 (Figure 4a, left, black line and symbols). Such a qualitative change was consistent with disordering of lipid chains and/or intercalation of the solute between the lipid chains at the hydrophobic/hydrophilic interface, *i.e.*, at site III (Figure 1c). Solute binding to site III was further implied by the fact that the maximum value of 110.0 Å^2^, which was attained by ⟨*A*⟩, was greater than the largest value of the cross-sectional area of disordered hydrocarbon chains in stable, solute-free surfactant bilayers – the observed maximum area is ≈41 Å^2^ per single chain,^7^ and theoretically predicted area is ≈44 Å^2^ per single chain.^42^

The maximum hydrocarbon region thickness *d*_*CH*_^*max*^ (case *ii*, all solute in the hydrocarbon region) initially decreased from 24.7 to 23.0 Å as benzyl alcohol concentration was increased to *R*_*S*_ ≈ 2.4, and further increased to 23.3 Å as concentration *R*_*S*_ of 3.0 was reached, Figure 4b, left (black line and symbols). In the initial decrease, shortening of the lipid chains due to disordering was manifested.

The minimum hydrocarbon region thickness *d*_*CH*_^*min*^ (case *i*, all solute in the headgroup region) decreased with solute concentration from 24.7 to 14.0 Å, indicating that the maximum average cross-sectional area attained by the lipid chain would be 109.6 Å^2^ (Figure 4b, left, black square symbols). Disordering of lipid chains can cause expansion of the lipid area, shortening of the lipid chains, and thinning of the hydrocarbon layer. However, such disordering is not expected to expand the lipid area to an extent, which would lead to greater average cross-sectional chain areas than the largest observed and theoretically predicted area of ≈41 and ≈44 Å^2^ per single chain. Thus, partitioning of benzyl alcohol solely into the polar headgroup region, *i.e.*, solely into site II, was not likely.

The expansion of bilayer area, suggested on the basis of behavior of ⟨*A*⟩, was expected to lead to thinning of interbilayer water layer at fixed hydration. The variation of water layer thickness with solubilization confirmed this assumption since both the minimum thickness *d*_*W*_^*min*^ (case *ii*, all solute in the hydrocarbon region) and the maximum thickness *d*_*W*_^*max*^ (case *i*, all solute in the headgroup region) of the water layer decreased monotonically as the solute concentration was increased (Figure 4c, left, black symbols and lines).

#### Behavior of Structural Parameters at Low Hydration

The behavior of the average surface area ⟨*A*⟩ and of the maximum and minimum hydrocarbon layer thickness (*d*_*CH*_^*max*^and *d*_*CH*_^*min*^) at the low hydration *R*_*W*_ of 8 shown in Figure 4a,b, right (black symbols and lines) was similar to the behavior of these parameters at the high hydration (previous section): ⟨*A*⟩ increased from 56.0 to 101.3 Å^2^; *d*_*CH*_^*max*^ decreases from 27.4 to 23.3 Å as solute concentration *R*_*S*_ was increased to 2.0 and increased to 23.6 Å when *R*_*S*_ was further increased to 2.5; *d*_*CH*_^*min*^ decreased monotonically from 27.4 to 15.1 Å, indicating the maximum average cross-sectional chain area of 101.6 Å^2^, Figure 4a,b, right (black symbols). Equally as in the high hydration case, we can conclude that (1) benzyl alcohol expanded the bilayer area and disordered hydrocarbon lipid chains; (2) the solute was most likely not distributed solely in the headgroup region of the bilayer (occupying thus the site II only), but was also partitioning into the hydrocarbon region (occupying thus the sites III, IV, and V); (3) some of the benzyl alcohol molecules were most likely accommodated at the hydrophobic/hydrophilic interface within the bilayer, *i.e*., site III was populated. The minimum as well as the maximum thickness of the interbilayer water layer (*d*_*W*_^*min*^ and *d*_*W*_^*max*^) was seen to decrease as solute concentration was increased, Figure 4c, right (black symbols and lines), which was the same qualitative change as in the bilayer systems of high hydration. The minimum water layer thickness *d*_*W*_^*min*^ was predicted to attain negative values when the solubilization *R*_*S*_ was greater than ≈1.0. This would be indicative of headgroup interdigitation.

### Characterization of the Lipid Molecule by NMR Spectroscopy

The effect of hydration on the ordering of hydrocarbon chains in solute-free DMPC bilayers at several values of hydration is described in the Supporting Information in section Characterization of Lipid Molecule by NMR Spectroscopy.

#### Effect of the Solute on Lipid Chain Order Profile in the Bilayer System of High Limited Hydration

**Figure 5:**
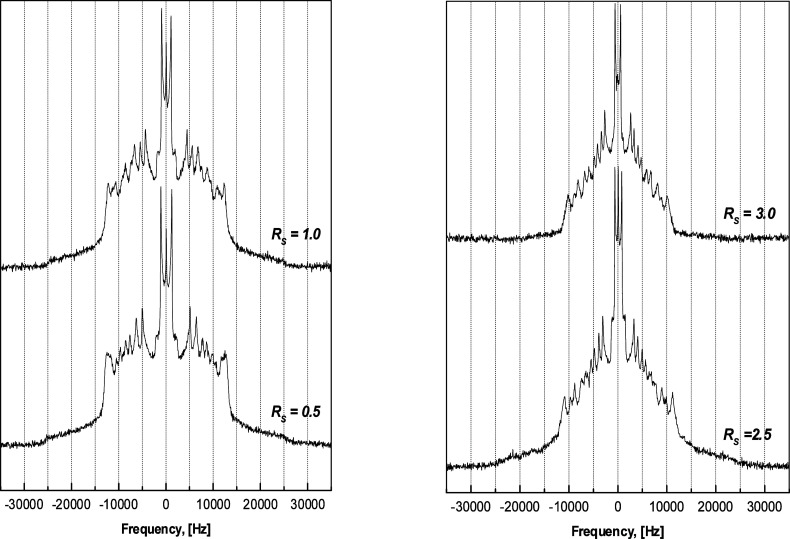
^2^H NMR spectra obtained on lipid chains in DMPC/water/benzyl alcohol system at the high hydration *R_W_* of 24 and temperature of 313 K. Adapted with permission from ref (53). Copyright 2000 University of Leeds and Šturcová.

**Figure 6:**
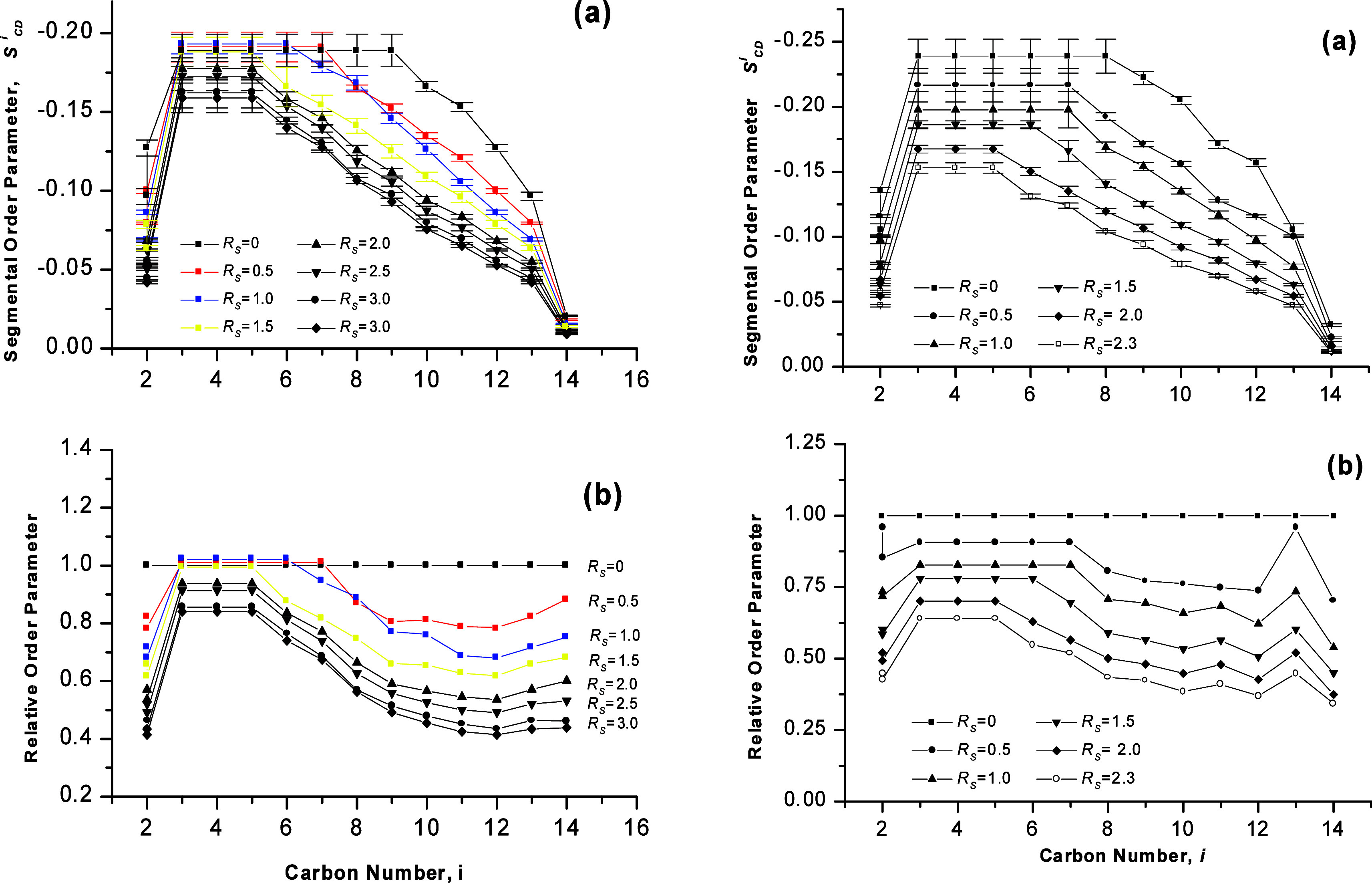
DMPC bilayers at various concentrations of benzyl alcohol *R_S_* and temperature of 313 K. Left: hydration *R_W_* = 24. Right: hydration *R_W_* = 8. (a) Segmental order profiles *S_CD_^i^* and (b) profiles of relative segmental order parameter. *S_CD_^i^* values corresponding to lipid chains in bilayers at *R_W_* = 22.9 were taken as the reference for the high hydration, and those at *R_W_* = 8.9 for the low hydration. Adapted with permission from ref (53). Copyright 2000 University of Leeds and Šturcová.

Typical ^2^H NMR spectra of the DMPC bilayer system with water to a lipid mole ratio *R*_*W*_ of 24 at temperature 313 K acquired at various solute concentrations *R*_*S*_ are shown in Figure 5, and the segmental *S*_*CD*_^*i*^ and the relative segmental order profiles are presented in Figure 6 left. Benzyl alcohol caused reduction of quadrupolar splitting Δν_*Q*_^*i*^ corresponding to the CD_2_ segment in position *i* in the lipid chain and consequently reduced chain order and, as is indicated by the relative segmental order parameters, the effect was greatest toward the center of the bilayer away from the headgroup region (*cf.*Figure S7 in Supporting Information).

**Figure 7:**
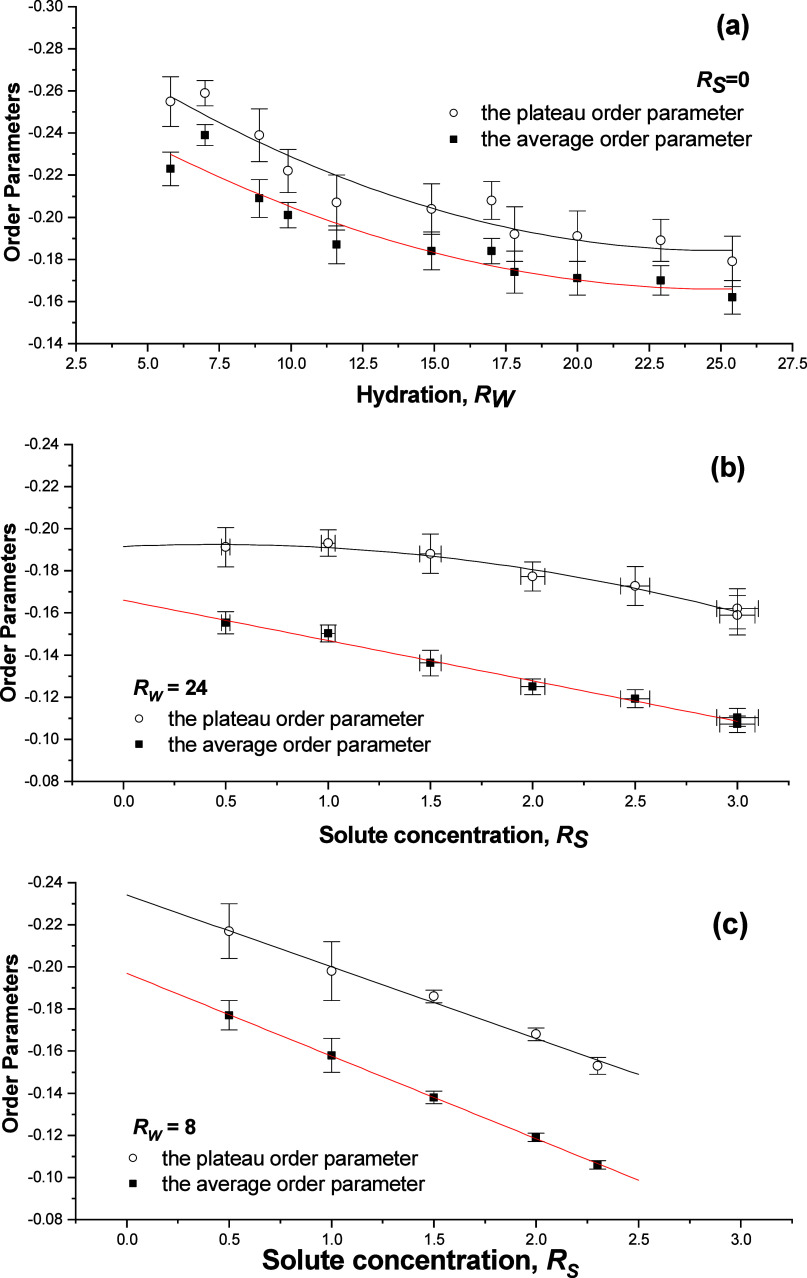
Variation of plateau *S_CD_^P^* and of average order parameter ⟨*S*⟩ (a) with hydration *R_W_* in solute-free DMPC bilayers; (b) with benzyl alcohol concentration *R_S_* at the high hydration *R_W_* of 24; (c) at the low hydration *R_W_* of 8; temperature of 313 K. Reprinted with permission from ref (53). Copyright 2000 University of Leeds and Šturcová.

The magnitude of the plateau order parameter *S*_*CD*_^*P*^ remained constant or changed only within the error of measurement for solubilizations *R*_*S*_ up to 1.5 and it decreased for higher solubilizations (Figure 6, left and Figure 7b). The magnitude of the average order parameter ⟨*S*⟩, defined by eq 1, varied linearly with *R*_*S*_ on the whole interval of benzyl alcohol concentrations studied at this hydration: the magnitude of ⟨*S*⟩ decreased from 0.155 at *R*_*S*_ = 0.5 to 0.107 at the solubility limit *R*_*S*_^*SL*^ of 3.0 with gradient of −0.019 (Figure 7b). Addition of benzyl alcohol to bilayers at this level of hydration also caused shortening of the length of the plateau region of the hydrocarbon chains: in the absence of any solute, the segmental order parameter *S*_*CD*_^*P*^ was constant between position *i* = 3 and position *i* = 9; in the presence of benzyl alcohol, the plateau began at the same carbon as in the solvent-free system, at *i* = 3, but shortened gradually with the solute concentration *R*_*S*_ until it reached the final length of three CD_2_ segments – *i.e.*, the end of the plateau is at carbon position *i* = 5. This final length of the plateau was attained at *R*_*S*_ of approximately 1.5 (Figure 6a, left).

*S*_*CD*_^*i*^ profiles indicated that for benzyl alcohol concentrations *R*_*S*_ up to 1.5, the steric constraints in the plateau region were maintained while the restrictions imposed on the remaining CD_2_ segments were relieved upon accommodation of the solute by the bilayer (Figure 6 left). This fact suggested that the solute molecules (all or fraction of them) must have been located at the hydrophobic/hydrophilic interface of the bilayer in site III, in which the hydroxyl group of benzyl alcohol molecules was suggested to interact with the carbonyl group of either hydrocarbon chain of the DMPC molecule and the aromatic ring was likely to be intercalated between the chains along the first few CD_2_ segments. Such a location (site) enables formation of free volume available to the remaining parts of the lipid chains in the bilayer center, which would be allowed to disorder. At the same time, site III places steric constraints on the first few segments and the order of these segments would not be expected to change significantly compared to solute-free bilayer. Adsorption of benzyl alcohol at the hydrophobic/hydrophilic interface agreed well with conclusions made on the basis of low-angle diffraction data (section Structural Parameters of DMPC Bilayer in Limited Hydration Regime as Determined by X-ray Diffraction – see above) and with Pohorille and Wilson^43^ who suggested that amphiphilic solutes tend to accumulate at the interface because they can simultaneously place their hydrophilic portions into the hydrophilic environment and their hydrophobic portions into the nonpolar environment. Alternatively, some of the qualitative change of *S*_*CD*_^*i*^ profiles for *R*_*S*_ ≤ 1.5 could be explained as follows: When López Cascales *et al.*(6) introduced benzyl alcohol into the center of the DMPC bilayer (our site V) at surgical concentrations (*R*_*S*_ ≈ 0.04) in molecular dynamics simulation studies, they found it to be located in a region populated by carbons *i* = 9 to *i* = 12 (*i.e.*, in the equivalent of site IV in this study) and they observed a significant decrease in *S*_*CD*_^*i*^ for positions *i* = 10 and *i* = 11.

The overall decrease of the order of CD_2_ segments (*S*_*CD*_^*i*^) along the full length of the hydrocarbon chain observed at benzyl alcohol concentrations *R*_*S*_ greater than 1.5 was the same qualitative change as the overall disordering of the lipid chains induced by increased hydration in the solute-free systems (section Effect of Hydration on the Hydrocarbon Chain Order Profile in Supporting Information). Since the change of the chain order with hydration *R*_*W*_ was caused by processes that take place in the headgroup region of the bilayer, it suggested that the overall reduction of *S*_*CD*_^*i*^ values upon addition of benzyl alcohol was caused by those solute molecules that were placed in the hydrophilic headgroup region (site II).

#### Structural Parameters of DMPC Bilayer in Limited Hydration Regime as Determined by NMR Spectroscopy and X-ray Diffraction: High Hydration

Apart from information on hydrocarbon chain order, ^2^H NMR can also be employed to study the dimensions of the lipid molecule. Using the values of the average order parameter ⟨*S*⟩, the average cross-sectional chain area projected onto the hydrophobic/hydrophilic interface *a*_*CH*_ was determined using the empirical model according to eq 7 with parameters 1/*a*_*CH*_^*e*^=0.057 Å^–2^_,_ 1/*a*_*CH*_^*max*^=0.002 Å^–2^, and *K*_*S*_ = 0.216. The parameters had been obtained by fitting DMPC bilayer data (Table S1) and two hypothetical data points to eq 7 – see previous sections. Values of *a*_*CH*_ were also obtained from a linear fit to DMPC data resulting in determination of parameters *p*_1_ = 0.019 Å^–2^ and *p*_2_ = 0.079 Å^–2^.

Following *a*_*CH*_ determination from NMR data according to the empirical exponential and linear models, the average cross-sectional area of the lipid molecule *a*_*L*_ was calculated assuming that *a*_*L*_ = 2*a*_*CH*_, Figure 4a, left (red line and symbols – exponential fit; green line and symbols – linear fit). Once the values of *a*_*L*_ were known, the thickness of the hydrocarbon region of the bilayer *d*_*CH*_ was obtained from NMR order parameters (Figure 4b, left, red and green line and symbols) using the following relationship:
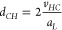
9where *v*_*HC*_ is the volume of the hydrocarbon portion of the lipid molecule, *i.e.*, it was assumed that the thickness of the hydrocarbon region is twice the average length of the lipid chain. In this case, using eq (S14) of the Supporting Information, the thickness of the bilayer can be also expressed as
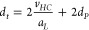
10where *d*_*P*_ is the thickness of the polar headgroup region.

The thickness of the interbilayer water layer *d*_*W*_ is another parameter of the mesoscopic structure that was determined using the combination of NMR and X-ray data: first, the thickness of the bilayer *d*_*t*_ was obtained from NMR data according to eq 10, and then eq (S15) of Supporting Information was used for *d*_*W*_ determination, where the values of the lamellar repeat distance *D* were obtained from low-angle X-ray diffraction (Tables S3 and S4, Supporting Information). The values of 767.2 Å^3^ for the volume of the hydrocarbon portion of the lipid molecule^36^*v*_*HC*_ and the value of 7.6 Å for the thickness of the headgroup region *d*_*P*_ were used in these calculations (Supporting Information).

The average cross-sectional lipid area *a*_*L*_ was seen to increase from 63.5 (62.3) Å^2^ in the solute-free bilayer to 85.0 (72.8) Å^2^ at a solute concentration *R*_*S*_ of 3.0 when the exponential (linear) fit was used for calculation (Figure 4a, left). The hydrocarbon region thickness *d*_*CH*_ was seen to decrease from 24.2 to 18.0 Å (exponential fit) or from 24.6 to 21.1 Å (linear fit) in the same concentration range (Figure 4b, left). The water layer thickness *d*_*W*_ was seen to decrease from 18.7 Å at *R*_*S*_ = 0.0 to 8.4 Å as the solute concentration *R*_*S*_ was increased to 2.5 and remained at approximately 8.5 Å for *R*_*S*_ up to 3.0 (exponential fit), *d*_*W*_ decreased from 18.3 Å to approximately 5.5 Å as *R*_*S*_ was increased from 0.0 to 3.0 and linear fit was used for calculation (Figure 4c, left).

The variation of *a*_*L*_ and *d*_*CH*_ reflected the observed disordering of the hydrocarbon lipid chains and the consequent decrease in the magnitude of ⟨*S*⟩, the average order parameter. Values of *a*_*L*_ were smaller than the values of the average surface area per lipid ⟨*A*⟩ that were estimated solely from X-ray diffraction data (Figure 4a, left). Such discrepancy indicated, that, apart from chain disordering, the bilayer area expanded also due to insertion of benzyl alcohol at the hydrophobic/hydrophilic interface in site III – this behavior agreed with conclusions made above based solely on X-ray diffraction data in previous sections.

The values of the hydrocarbon region thickness *d*_*CH*_ were greater than the minimum estimate of this thickness *d*_*CH*_^*min*^ based on X-ray diffraction data under the assumption that all of the solute molecules were placed in the headgroup region in site II (see above and Supporting Information). This showed that benzyl alcohol molecules did not partition solely into the headgroup region (site II) but that they also partitioned into the nonpolar hydrocarbon region of the bilayer (sites III and IV). At the same time, the values of the hydrocarbon region thickness *d*_*CH*_ were smaller than the maximum estimate of this thickness *d*_*CH*_^*max*^ based on X-ray diffraction data under the assumption that all solute molecules were accommodated by the hydrocarbon region of the bilayer (in the sites III, IV, and V). This indicated that benzyl alcohol molecules partitioned also into the headgroup region of the bilayer.

One has to bear in mind that the real hydrocarbon layer thickness would be greater than the thickness *d*_*CH*_ determined from ^2^H NMR measurements in the case benzyl alcohol created a layer in the center of the bilayer, *i.e.*, if it occupied site V, and it would mean that no benzyl alcohol was placed in the headgroup region in site II. However, the qualitative behavior of *S*_*CD*_^*i*^ profiles of the hydrocarbon chains described above (the decrease in *S*_*CD*_^*i*^ along the full length of the hydrocarbon chain) proved that the solute was indeed partitioning into the headgroup region (site II) apart from partitioning into the hydrocarbon region (sites III, IV, and V).

The thinning of water layer thickness *d*_*W*_ for solute concentrations up to 2.5 (exponential fit) or up to 3.0 (linear fit) was consistent with expansion of the bilayer surface area. When an exponential fit was used, *d*_*W*_ did not change significantly as *R*_*S*_ was increased from 2.5 to 3.0, while the bilayer area increased in this concentration range – the water layer thickness was most likely kept constant by those water molecules that were displaced from the headgroup region by benzyl alcohol molecules occupying site II.

#### Effect of the Solute on the Lipid Chain Order Profile in the Bilayer System of Low Hydration

^2^H NMR spectra of DMPC bilayers with low hydration of *R*_*W*_ = 8 at various concentrations of benzyl alcohol are shown in Figure S8. The segmental *S*_*CD*_^*i*^ and the relative segmental order profiles of DMPC bilayer systems with a water to lipid mole ratio *R*_*W*_ of 8 at temperature 313 K and at various benzyl alcohol concentrations are presented in Figure 6 (right).

Benzyl alcohol reduced chain order, and equally as in bilayers of high hydration *R*_*W*_ = 24, the relative segmental order parameters indicated that the disordering effect was greatest for the chain-end segments of the chains. The magnitude of the plateau order parameter *S*_*CD*_^*P*^ decreased linearly with solute concentration *R*_*S*_ from 0.217 at *R*_*S*_ = 0.5 to 0.153 at *R*_*S*_ = 2.3 and the gradient of this linear variation was −0.034 (Figure 7c). The decrease of the magnitude of the average order parameter ⟨*S*⟩ with *R*_*S*_ (Figure 7c) can also be described as linear: as *R*_*S*_ was increased from 0.5 to 2.3, the magnitude of ⟨*S*⟩ dropped from 0.177 to 0.106 with the gradient of −0.039. Upon addition of benzyl alcohol to DMPC bilayers, the length of the plateau region was reduced: In the solute-free system, the plateau spanned CD_2_ segments between positions *i* = 3 to *i* = 8; however, when benzyl alcohol was dissolved in the bilayer, the plateau region began at the same position as in solute-free system – at *i* = 3 – but the length of the region was reduced gradually and at *R*_*S*_ of 2.0, the plateau ended at the segment in position *i* = 5 (Figure 6 right). As the solute concentration was further raised to 2.3, the order of the plateau segments *S*_*CD*_^*P*^ decreased, but there were no further changes in the length and position of the plateau region. Thus, the final length and location of this region was the same as in bilayers containing benzyl alcohol at the high limited hydration of 24. However, the benzyl alcohol content of *R*_*S*_ ≈ 2.0, at which the final length of the plateau was attained, differed from the high hydration value of ≈ 1.5.

The overall reduction of *S*_*CD*_^*i*^ values observed at any benzyl alcohol concentration used at the low hydration indicated that the lipid headgroups were moved further apart by those benzyl alcohol molecules that were accommodated in the polar headgroup region in site II.

Site III was identified in bilayers of the high and low hydration on the basis of X-ray data (see above), this site was confirmed to exist for the highly hydrated bilayers also by ^2^H NMR. Benzyl alcohol molecules in site III are expected to create free volume for the nonplateau CD_2_ segments and at the same time to place steric constraints on the plateau segments. Since the relative order parameter of the plateau CD_2_ segments was greater than the relative order parameter of the remaining segments (Figure 6 right) at any solute concentration in the bilayers at *R*_*W*_ = 8, it was clear that the steric constraints in the plateau region were indeed relieved less than the constraints for the rest of the chain segments. This was very likely caused by benzyl alcohol localized in site III or if reasoning based on observations of López Cascales *et al.*(6) was used, it could also indicate binding to site IV.

#### Structural Parameters of the DMPC Bilayer in the Limited Hydration Regime as Determined by NMR Spectroscopy and X-ray Diffraction: Low Hydration

Using the values of the average order parameter ⟨*S*⟩, the average cross-sectional area of lipid molecule *a*_*L*_*,* the thickness of the hydrocarbon region *d*_*CH*_, and the thickness of the interbilayer water layer *d*_*W*_ were determined in the way described for the high hydration case above. Variation of these mesoscopic structural parameters with solute concentration *R*_*S*_ is shown in Figure 4 right (red lines and symbols – values obtained using exponential fit; green lines and symbols – values obtained using linear fit). The average cross-sectional lipid area *a*_*L*_ was seen to increase from 57.3 Å^2^ at a solute concentration *R*_*S*_ of 0.0 to 85.7 Å^2^ at *R*_*S*_ of 2.3 (exponential fit) or to increase from 57.9 to 73.1 Å^2^ (linear fit), Figure 4a, right. The hydrocarbon region thickness *d*_*CH*_ was seen to decrease from 26.8 to 17.9 Å (exponential fit) or from 26.6 to 21.0 Å (linear fit) in the same concentration range (Figure 4b, right). The water layer thickness *d*_*W*_ was seen to decrease from 6.2 Å at *R*_*S*_ = 0.0 to 0.5 Å at solute concentration of 1.5 and after that to increase to 2.1 Å at *R*_*S*_ of 2.3 (exponential fit); when a linear fit was used, *d*_*W*_ decreased from 6.5 Å at *R*_*S*_ = 0.0 to −1.4 Å at *R*_*S*_ = 2.0 and after that increased to −1.0 Å at *R*_*S*_ = 2.3 (Figure 4c, right). The variation of *a*_*L*_ and *d*_*CH*_ reflected the disordering of the hydrocarbon lipid chains described in terms of the decrease of the segmental, the plateau, and the average order parameter *S*_*CD*_^*i*^, *S*_*CD*_^*P*^, and ⟨*S*⟩, respectively.

Values of the average cross-sectional lipid area *a*_*L*_ determined from NMR data were smaller than the values of the average surface area per lipid ⟨*A*⟩ determined from X-ray data, which indicated that, apart from chain disordering, the bilayer area expanded also due to insertion of benzyl alcohol at the hydrophobic/hydrophilic interface in site III. This agreed with conclusions based solely on X-ray diffraction data. Equally, as for the high hydration, comparison of *d*_*CH*_ obtained from NMR data with the minimum and the maximum estimates of the hydrocarbon layer thickness obtained from the X-ray data *d*_*CH*_^*min*^ and *d*_*CH*_^*max*^ (Figure 4b, right) indicated partitioning of benzyl alcohol molecules into both the headgroup (site II) and the hydrocarbon (sites III and IV) regions of the bilayer. The thinning of the water layer thickness *d*_*W*_ for solute concentrations up to 1.5 (exponential fit) or up to 2.0 (linear fit) was consistent with expansion of the bilayer surface area. Values of *d*_*W*_ increase when *R*_*S*_ is increased from 1.5 (2.0) to 2.3, while the bilayer area is seen to increase in this concentration range – this is most likely due to those water molecules that were displaced from the headgroup region by benzyl alcohol molecules occupying site II. If the linear fit was justified by physics arguments, negative values of *d*_*W*_ obtained from the linear fit would be indicative of headgroup interdigitation.

When quantification of distribution of benzyl alcohol within the bilayer was performed (Supporting Information, section Quantification of Distribution of Benzyl Alcohol within the Bilayer), further support was found for the conclusions about hydration and solute concentration dependence of the population distribution of benzyl alcohol in a DMPC bilayer.

#### Limiting Behavior of the Lipid Chains in Bilayer Systems as Detected by Deuterium NMR Spectroscopy

**Figure 8:**
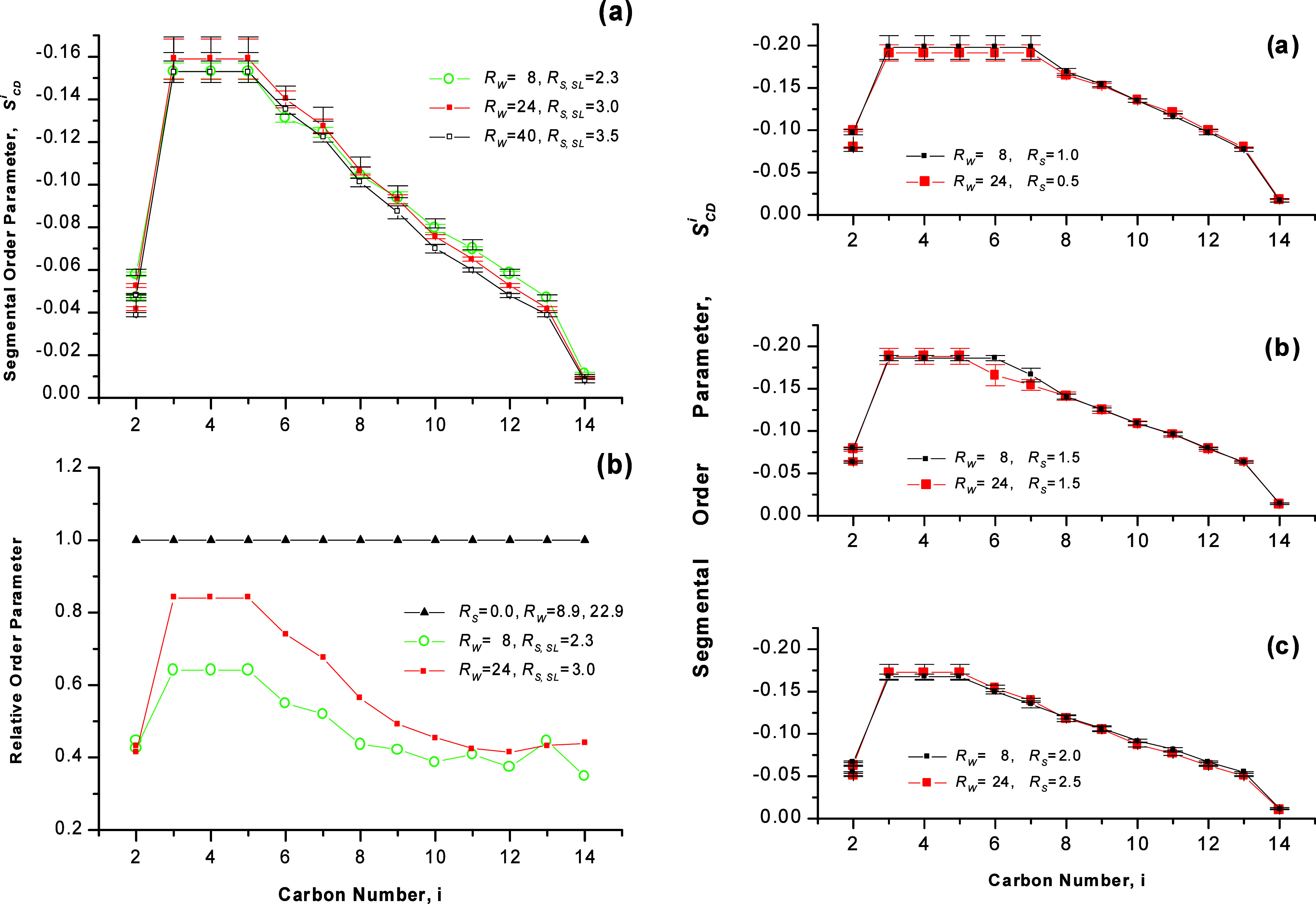
Left: (a) The limiting segmental order parameter profiles and (b) the limiting profiles of relative segmental order parameter of the lipid chains in DMPC bilayers at hydration *R_W_* of 8, 24, and 40 and with benzyl alcohol concentration at the respective solubility limits *R_S_^SL^* and temperature of 313 K. Right: Segmental order parameter profiles at the low *R_W_* = 8 and at the high *R_W_* = 24 hydration chosen so that the values of the average order parameter ⟨*S*⟩ and the profiles were almost identical. This is attained by combination of hydration *R_W_* with suitable concentration of benzyl alcohol *R_S_*: (a) ⟨*S*⟩ = −0.158 at *R_W_* = 8, *R_S_* = 1.0 compared to ⟨*S*⟩ = −0.155 at *R_W_* = 24, *R_S_* = 0.5; (b) ⟨*S*⟩ = −0.138 at *R_W_* = 8, *R_S_* = 1.5 compared to ⟨*S*⟩ = −0.136 at *R_W_* = 24, *R_S_* = 1.5; (c) ⟨*S*⟩ = −0.119 at *R_W_* = 8, *R_S_* = 2.0 compared to ⟨*S*⟩ = −0.119 at *R_W_* = 24, *R_S_* = 2.5. Adapted with permission from ref (53). Copyright 2000 University of Leeds and Šturcová.

The impact of benzyl alcohol on the segmental order parameter profiles of lipid chains was studied at the limit of its solubility at three different hydrations – at water to lipid mole ratio *R*_*W*_ of 8, 24, and 40, temperature was 313 K. It was clearly observed, that *S*_*CD*_^*i*^ profiles were almost identical when the content of benzyl alcohol in the bilayer *R*_*S*_ was at the corresponding solubility limit *R*_*S*_^*SL*^ at each hydration (Figure 8a, left) – such an observation had not been reported before for any lipid bilayer system according to the author’s knowledge. It is also noticeable, that there was very little variation in the value of the average order parameter at the solubility limit at the corresponding hydration denoted as ⟨*S*_*r*_^*SL*^⟩ (Table 2). Such findings suggest that a bilayer cannot disorder beyond a certain “limiting” profile of segmental order parameters and this profile is identical with *S*_*CD*_^*i*^ profiles presented in Figure 8a, left.

**Table 2:** Hydration Dependence of Gradient *k_W_*^f^

**hydration**, ***R_W_***	**solubility limit**, ***R**_**S**_^**SL**^*	|⟨*S*^0^⟩|^a^	|⟨*S*^*SL*^⟩|^b^	|⟨*S*_*r*_^*SL*^⟩|^b^	**gradient *k_W_*^c^**	*a*_CH_^*SL*^ [Å^2^]^d^
8	2.3	0.197	-	0.106	0.43	42.86
	(2.5)^e^	(0.197)	(0.099)	-	(0.40)	(45.33)
24	3.0	0.166	0.109	0.110	0.33	41.76
	3.0	0.166	0.109	0.107		42.58
40	3.5	-	-	0.107	0.29	42.58

a ⟨*S*^0^⟩ is the average order parameter in solvent-free bilayers found by regression analysis.

b ⟨*S^SL^*⟩ and ⟨*S_r_^SL^*⟩ are the average order parameter found by regression analysis and experimentally, respectively, in bilayers with benzyl alcohol concentration at the solubility limit at the corresponding hydration.

c The values of the gradient *k_W_* of the change of the ε-value with *R_S_* calculated using eq 18 are presented.

d The values of the average cross-sectional area of the hydrocarbon chain *a_CH_^SL^* in bilayers at *R_S_^SL^* are calculated using values of |⟨*S_r_^SL^*⟩| and eq 7.

e At the hydration of 8, there are two values of the benzyl alcohol concentration stated as the solubility limit value *R_S_^SL^*: the value in the parentheses is a value determined by the ^31^P NMR method, the other value is a benzyl alcohol concentration, which was used in a ^2^H NMR experiment chosen, so that is was close to the respective solubility limit, but the excess benzyl alcohol phase was avoided. If a value of a certain parameter was not determined at given *R_W_*, sign “-” is used.

f Table reprinted with permission from ref (53). Copyright 2000 University of Leeds and Šturcová.

The profiles of the relative segmental order parameter are shown in Figure 8b, left, and it can be seen that benzyl alcohol had the greatest effect at the lowest hydration studied *R*_*W*_ of 8 – as hydration was increased, the relative order parameter of the individual segments increased, the impact of benzyl alcohol decreased.

Further, it was found that the effect of hydration and the effect of suitable amount of benzyl alcohol added to the bilayer could be combined to give identical segmental order parameter profile under different conditions: at the low hydration *R*_*W*_ = 8 and at benzyl alcohol concentration *R*_*S*_ of 1.0, 1.5, and 2.0, the hydrocarbon chains adopted almost identical *S*_*CD*_^*i*^ profiles (and thus also almost the same values of the average ⟨*S*⟩ and the plateau *S*_*CD*_^*P*^ order parameters) as at the high hydration *R*_*W*_ = 24 and at benzyl alcohol concentration *R*_*S*_ of 0.5, 1.5, and 2.5, respectively (Figure 8 right).

Variation of the modulus of the average order parameter|⟨*S*⟩| with solute concentration *R*_*S*_ can be described as linear both at low *R*_*W*_ = 8 and high *R*_*W*_ = 24 (Figure 7b,c and Figure S11a):
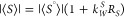
11where |⟨*S*^0^⟩| and *k*_*W*_^*S*^ are the value of the average order parameter in the solute-free system and the value of the gradient, respectively, found by regression analysis.

It follows from eq 11 for variation of the relative average order parameter  with *R*_*S*_ that
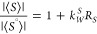
12where *k*_*W*_^*S*^ is the gradient of the change of the relative order parameter  with *R*_*S*_ at the level of hydration given by *R*_*W*_ (Figure S11b). It is valid that
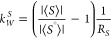
13

When the value of the average order parameter |⟨*S*_*r*_^*SL*^⟩| obtained experimentally at the solubility limit *R*_*S*_^*SL*^ and the value of the average order parameter obtained experimentally in the solute-free system |⟨*S*_*r*_^0^⟩| are used, then
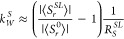
14

It is possible to introduce expression
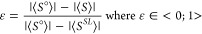
15

Equation 15 defines a new quantity ε, which expresses a change of the modulus of the average order parameter attained at the solute content of *R*_*S*_ relative to the maximum change of the modulus of the average order parameter at a given hydration – that is a change of the modulus of the average order parameter attained at the solubility limit *R*_*S*_^*SL*^.

**Figure 9:**
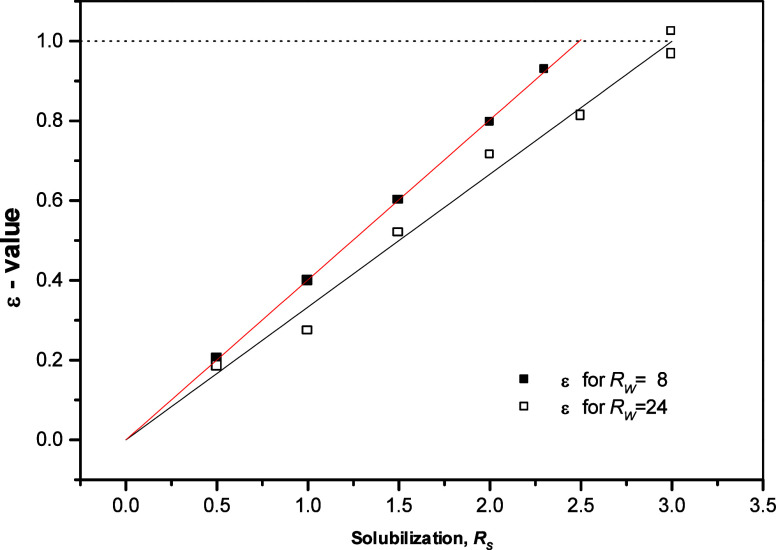
Dependence of the ε-value on the concentration of benzyl alcohol *R_S_* at low hydration *R_W_* = 8 and high hydration *R_W_* = 24. Reprinted with permission from ref (53). Copyright 2000 University of Leeds and Šturcová.

The dependence of the ε-value on *R*_*S*_ is linear at hydrations *R*_*W*_ of 8 and 24 (Figure 9) is the following:
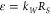
16

The gradient *k*_*W*_ of the dependence of the ε-value on *R*_*S*_:
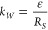
17and for values obtained experimentally (for ε = 1 and *R*_*S*_ = *R*_*S*_^*SL*^):
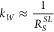
18

The gradient *k*_*W*_ varies with hydration *R*_*W*_: the solubility limit *R*_*S*_^*SL*^ was observed to increase with increasing hydration (see above). Therefore, using eq 18, the gradient *k*_*W*_ decreased with increasing hydration *R*_*W*_ from 0.43 at *R*_*W*_ = 8 to 0.29 at *R*_*W*_ = 40 (Table 2). The decrease can also be seen in the behavior of the ε-value vs *R*_*S*_ in Figure 9: the value of *k*_*W*_ found by fitting was 0.40 for *R*_*W*_ = 8, and it was 0.33 for *R*_*W*_ = 24 – this observation corresponded to the behavior of values of *k*_*W*_ presented in Table 2. The decrease of the gradient *k*_*W*_ with hydration *R*_*W*_ indicated that the disordering effect of benzyl alcohol on the lipid chain was attenuated by increased hydration of the bilayers, *i.e.*, addition of the same amount of benzyl alcohol to the bilayers at the high hydration *R*_*W*_ = 24 would result in a smaller relative change (the smaller ε-value) than the same addition at the low hydration *R*_*W*_ = 8.

Values of *k*_*W*_^*S*^ – the gradient of the change of the relative average order parameter  with *R*_*S*_ at given hydration – were calculated for selected values of hydration *R*_*W*_ using values presented in Table S5 (Supporting Information) and eq 14. The result of this calculation is presented in Table S5 (Supporting Information). The values of *k*_*W*_^*S*^ determined from linear fits are −0.199 and −0.116 at hydration *R*_*W*_ of 8 and 24, respectively (Figure S11b). These two *k*_*W*_^*S*^ values found by fitting compare favorably with the corresponding values of *k*_*W*_^*S*^ calculated using eq 14 (Table S5). The calculated *k*_*W*_^*S*^ values were hydration dependent and they were fitted to a polynomial of second order:
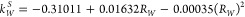
19

It can be seen, according to eqs 11 and 19, that the average order parameter ⟨*S*⟩ is dependent both on hydration *R*_*W*_ and on benzyl alcohol content *R*_*S*_:
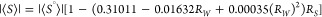
20

It means that we can determine benzyl alcohol concentration *R*_*S*_ needed to attain required average order parameter ⟨*S*⟩ at given hydration *R*_*W*_. This is based on the observations presented, which implied that ⟨*S*⟩ is dependent both on hydration *R*_*W*_ and solute concentration *R*_*S*_. Further, it implied that the same order parameter profile and ⟨*S*⟩ can be reached by combination of these two factors (*R*_*W*_ and *R*_*S*_) and that the same limiting *S*_*CD*_^*i*^ profile determines when the incorporation of the solute into the bilayer is terminated – *i.e*., when the solubility limit *R*_*S*_^*SL*^ is reached, at each hydration studied. Using eq 20, we have calculated concentrations of benzyl alcohol *R*_*S*_ required to obtain several selected values of ⟨*S*⟩ at a given level of hydration *R*_*W*_. The results of this calculation are presented in Table S6 (Supporting Information). Table S7 (Supporting Information) contains calculated *R*_*S*_ values at hydration of 8 and 24 for specified |⟨*S*⟩| values taken from Table S6. These *R*_*S*_ values are compared with actual benzyl alcohol concentrations *R*_*S*_ used in those experiments in which the specified magnitude of the average order parameter ⟨*S*⟩ was reached (Table S7); the respective profiles of segmental order parameters *S*_*CD*_^*i*^ are in Figure 8 right. It is necessary to recall that the experiments at the two (*R*_*W*_ = 8 and *R*_*W*_ = 24) hydrations were not initially aimed at obtaining the almost identical *S*_*CD*_^*i*^ profiles presented in Figure 8 (right). The concentration of water *R*_*W*_ was accidentally combined with benzyl alcohol concentration *R*_*S*_, which enabled observation of very similar *S*_*CD*_^*i*^ profiles. It is possible to perform experiments under some of the conditions (hydration and solute concentration) given in Table S6 (Supporting Information), which would further verify and confirm results of this section, namely, the fact that a suitable combination of *R*_*W*_ and *R*_*S*_ values results in identical *S*_*CD*_^*i*^ profiles and that the same limiting profile of segmental order parameters is reached at each hydration as well as it would confirm the validity of eq 11 and consequently eq 12, eq 17, and eq 20.

## Discussion

### Interaction of Benzyl Alcohol with Lipid Bilayers

Addition of benzyl alcohol to DMPC bilayers both at low limited hydration *R*_*W*_ = 8 and high limited hydration *R*_*W*_ = 23 (X-ray) or *R*_*W*_ = 24 (NMR) caused expansion of the bilayer area that was reflected in the increase of the average area per lipid ⟨*A*⟩ (Figure 4). Two mechanisms causing the area expansion were identified: (1) Disordering of the lipid chains that resulted in reduced magnitude of the segmental order parameters *S*_*CD*_^*i*^ (Figure 6) and in an increase of the average cross-sectional chain (lipid) area *a*_*CH*_ (*a*_*L*_) and decrease of the bilayer and hydrocarbon layer thickness (Figure 4). (2) Insertion of the solute molecule at the hydrophobic/hydrophilic interface in site III that was indicated by the fact that *a*_*L*_ values were smaller than ⟨*A*⟩ values (Figure 4).

Our observation of benzyl alcohol disordering lipid chains at both hydrations (*R*_*W*_ = 8 and *R*_*W*_ = 24 at temperature of 313 K) aligned well with observations made by Turner and Oldfield.^45^ These authors observed no change in the order of the CD_2_ segment in position *i* = 6 and in bilayer thickness for a surgical level of concentrations (*R*_*S*_ ≤ 0.1), but the order of this segment decreased for *R*_*S*_ values up to 3.0. Turner and Oldfield also saw a decrease of order for all chain CD_2_ segments at *R*_*S*_ = 3.0 in DMPC vesicles at 311.15 K. The decreased chain order at *R*_*S*_ = 3.0 caused reduction of the bilayer thickness by 1.4 Å. Pope *et al.*(3) observed disordering of the CD_2_ segment in position *i* = 14 in DMPC/water/benzyl alcohol bilayers at *R*_*S*_ = 0.5 and temperature of 310.15 K.

Benzyl alcohol molecules accommodated themselves in various binding sites within the lipid bilayer: If the solute molecules were located in the headgroup region (consisting of the headgroup and the glycerol backbone), they contributed to the volume of the headgroup region and these binding sites were called site II (Figure 1b). If the solute molecules were placed in the hydrocarbon region contributing to the volume of this region, these sites for the binding of the solute were called site III, IV, and V (Figure 1c–e). We have proven that benzyl alcohol was binding simultaneously into the headgroup and into the hydrocarbon region of the bilayer (*e.g.*, Figure 4). Site II binding was confirmed to take place by ^2^H NMR of the lipid chains: benzyl alcohol molecules in the headgroup region force the lipid headgroups further apart loosening thus the lipid chain packing, and this was reflected in the overall decrease of the segmental order parameters *S*_*CD*_^*i*^ along the full length of the lipid chain in bilayers at the high and the low hydration (Figure 6). Parameters of the bilayer mesoscopic structure were calculated from ^2^H NMR data (by the use of the average order parameter ⟨*S*⟩ and eq 7 or a linear dependence, in combination with eq 9. Comparison of these parameters showed that *d*_*CH*_ values from NMR were greater than *d*_*CH*_^*min*^ values from X-ray, which provided evidence that the hydrocarbon sites (sites III or IV) for benzyl alcohol binding existed both at the high and the low hydration (Figure 4). Comparison of the thickness *d*_*CH*_ with the maximum hydrocarbon layer thickness *d*_*CH*_^*max*^ (which is a thickness of the hydrocarbon region when all of the solute molecules are placed in this region) showed that *d*_*CH*_ values were smaller both at low and high hydration. With the assumption that no benzyl alcohol molecules were accumulated in site V, this indicated that benzyl alcohol partitions also into site II. The evidence for existence of site III was obtained from comparison of the values of the average area per lipid ⟨*A*⟩ with the average cross-sectional area of lipid *a*_*L*_ at both the high and the low hydration (Figure 4). When a benzyl alcohol molecule was adsorbed at the hydrophobic/hydrophilic interface in site III in such way that its hydrophobic aromatic ring was inserted between the plateau regions of the hydrophobic hydrocarbon chains, the steric constraints in the plateau region were relieved less than in the chain end region and this was reflected in the greater relative segmental order parameter in the plateau region of the hydrocarbon chains (Figure 6).

The existence of binding sites in the headgroup region of DMPC bilayers that was proven in this work (site II) is consistent with the conclusions of Pope *et al.*,^3^ Boden *et al.*,^4,7^ and of Turnbull^22^ who worked in the limited hydration regime and above the gel–liquid crystal phase transition temperature and they assumed that benzyl alcohol resided in the headgroup region.

Apart from the current study, in which the existence of the site III was documented, some kind of interfacial binding site for benzyl alcohol was also proposed to exist by Turnbull^22^ and for amphiphilic solutes by Pohorille and Wilson.^43^

Existence of a site for binding of benzyl alcohol in the center of the hydrocarbon region (site V) cannot be proven or disproven on the basis of current data. According to theory of Marqusee and Dill^5^ on mixing solutes with bilayers, site V can be expected to exist and some perturbation to the chain order would thus be expected. However, López Cascales *et al.*(6) introduced benzyl alcohol into the center of the bilayer (the equivalent of site V) in molecular dynamics simulation studies and found it to be located in the region populated by carbons *i* = 9 to *i* = 12, which is equivalent of site IV, not the initial site V (Figure 1d). This would suggest that site V is not significantly populated. Further, these authors observed significant increase in disorder of the segments in position *i* = 10 and *i* = 11. One has to bear in mind that the effect of site IV (disordering of chain end segments) might be obscured by the effect of site III. Stouch and Bassolino^54^ also observed disordering when drug analogue nifedipine was present in the chain end region. On the other hand, Barry and Gawrisch^47^ found that some solutes (*e.g.*, alkanes and alcohols including octanol and decanol) that incorporated into the hydrocarbon region of the bilayer (DMPC, DOPC, DPPC) had very little or no observable effect on bilayer order.

At each hydration and solute concentration, there is a dynamic equilibrium between the individual sites – the benzyl alcohol molecules are presumed to exchange between the binding sites (sites II, III, IV, and V) both in the headgroup and in the hydrocarbon region. The concentration of the solute in the individual sites was shown to be dependent on hydration *R*_*W*_ and solute concentration *R*_*S*_: for *R*_*S*_ ≥ 1.5 and on condition that site V was not populated, the fraction of benzyl alcohol molecules in site III and IV (*X*_*S*_^*HC*^) was greater at the high hydration *R*_*W*_ = 23 than at the low hydration *R*_*W*_ = 8 and the fraction decreased with *R*_*S*_ at both hydrations (Figure S8), *i.e.*, population of site II increased with decreased hydration and with increased solute concentration. At high solute concentrations (*R*_*S*_ ≥ 2.5 at *R*_*W*_ = 24 and *R*_*S*_ ≥ 1.5 or *R*_*S*_ ≥ 2.0 at *R*_*W*_ = 8; Figure 7c), the water molecules were displaced from the headgroup region by benzyl alcohol molecules occupying site II. Those water molecules that were displaced by the benzyl alcohol molecules occupying site II contributed to the interbilayer water thickness *d*_*W*_ and prevented the increase of repulsive interbilayer forces, which would otherwise oppose bilayer surface expansion. At high hydration *R*_*W*_ = 24, the displacement of water molecules was indicated only when exponential dependence of the surface density of chains on the average order parameter was used for calculation, *i.e.*, when eq 7 was used. At low hydration *R*_*W*_ = 8, negative values of water layer thickness *d*_*W*_ obtained from linear dependence would be indicative of headgroup interdigitation for *R*_*S*_ > 1.0, while *d*_*W*_ values obtained from exponential dependence, eq 7, did not predict interdigitation of headgroups. Additional information is necessary to decide which of the two predicted behaviors actually take place. However, the exponential dependence is thought to describe behavior of the surface density of chains on a wider range of the average order parameter values better than the linear dependence. Therefore, it is likely that behavior predicted using eq 7 is more likely, *i.e.*, that displacement of water molecules at *R*_*W*_ = 24 is likely while no headgroup interdigitation at *R*_*W*_ = 8 is probable.

It has also been observed that it was possible to attain almost identical segmental order parameter *S*_*CD*_^*i*^ profiles for different compositions of benzyl alcohol containing DMPC bilayers – the effects of hydration *R*_*W*_ and solute concentration *R*_*S*_ combined to give almost identical shape of *S*_*CD*_^*i*^ profiles, Figure 8, and consequently, identical values of the average order parameter. On the basis of the experimental data, it was possible to determine a relationship, eq 20, for variation of the magnitude of the average order parameter |⟨*S*⟩| with the solute concentration *R*_*S*_ and hydration *R*_*W*_. It has been found that the maximum concentration of benzyl alcohol that can be accommodated by a DMPC bilayer, *i.e.*, the solubility limit *R*_*S*_^*SL*^, was hydration-dependent: it increased from *R*_*S*_^*SL*^ = 2.5 for 8 ≤ *R*_*W*_ ≤ 10 to *R*_*S*_^*SL*^ = 3.0 for 13 ≤ *R*_*W*_ ≤ 25 and it increased further to *R*_*S*_^*SL*^ = 3.5 as the hydration was increased to *R*_*W*_ = 40. As a result of the increase of *R*_*S*_^*SL*^ with increased *R*_*W*_, the gradient of the change of the ε-value with *R*_*S*_ (*k*_*W*_) decreased with increased hydration, eq 18, Table 2, and Figure 9. It means that increased hydration attenuated the effect of benzyl alcohol on the ordering state of the hydrocarbon chains in DMPC bilayers.

The observation that benzyl alcohol molecules were distributed between sites II, III, IV, and possibly V in a dynamic equilibrium, and the observation that the solubility limit *R*_*S*_^*SL*^ decreased as bilayer hydration was decreased were in qualitative agreement with the principal predictions of the lattice theory for solute partitioning into interfacial phases of chain molecules developed by Marqusee and Dill:^5^ (1) Apart from the hydrocarbon sites in the center of the bilayer (sites IV and V), the interfacial sites (the site III) also exist because benzyl alcohol has higher affinity for external solvent (water) than the hydrocarbon chain segments. (2) A decrease of hydration leads to increased surface density of the hydrocarbon chains; thus, according to the theory, the solute is expelled from the hydrocarbon region, which probably leads to lower solute concentrations that can be accommodated by the bilayer, *i.e.*, it leads to lower solubility limit values. The increased surface density upon dehydration explains also the fact that the distribution of benzyl alcohol in the lipid bilayer is hydration-dependent – *X*_*S*_^*HC*^ values (the values of the fraction of benzyl alcohol placed in site III and IV) were smaller at the low hydration *R*_*W*_ = 8 than at the high hydration *R*_*W*_ = 23 for *R*_*S*_ ≥ 1.5. Surface density of the lipid chains can be increased also by reduction of temperature: Boden *et al.*(4) worked on DMPC/water/benzyl alcohol systems at similar high hydration, *R*_*W*_ = 25, but at a lower temperature of 303.15 K. The effect of benzyl alcohol on the segmental order parameters *S*_*CD*_^*i*^ profiles was to cause overall disorder of CD_2_ segments along the full length of the hydrocarbon chains and to shorten the plateau region. Further, the plateau segments were disordered to a lesser extent than the chain end segments – this was the same behavior as observed at the higher temperature of 313 K for both the high *R*_*W*_ = 24 and the low *R*_*W*_ = 8 hydration in this work. However, the plateau order parameter *S*_*CD*_^*P*^ did not remain constant for any benzyl alcohol concentration used by Boden *et al.*(4) – this is a behavior different from that described in this work for the high hydration *R*_*W*_ = 24 at 313 K while it is a behavior similar to that described for the low hydration *R*_*W*_ = 8 at 313 K. A decrease in temperature leads to more ordered hydrocarbon chains, *i.e.*, to higher surface density of chains, which causes solute expulsion from the hydrocarbon region of DMPC bilayers in accordance with Marqusee and Dill.^5^ The solute expulsion might account for the fact that benzyl alcohol molecules were observed to accommodate themselves solely in the headgroup region and not in the hydrocarbon region at high hydration *R*_*W*_ = 25 at a temperature of 303.15 K by Boden *et al.*,^4,7^ while in the current work, binding sites in the headgroup as well as in the hydrocarbon region were confirmed to exist at the high hydration *R*_*W*_ = 24 or *R*_*W*_ = 23 and at a temperature of 313 K. We also showed that temperature, in other words, surface density of chains, most likely affected also the maximum concentration of benzyl alcohol that can be accommodated by a DMPC bilayer since the solubility limit *R*_*S*_^*SL*^ at hydration *R*_*W*_ of 40 decreased from 3.5 at 313 K to 3.0 at 298 K.

Limiting the segmental order parameter *S*_*CD*_^*i*^ profile of a hydrocarbon chain in a bilayer aggregate is proposed to exist here, and the profile is shown in Figure 8a, left. Independent of hydration, the hydrocarbon chains adopt this profile whenever the solute concentration is equal to the solubility limit *R*_*S*_^*SL*^ at the given hydration. Such behavior is expected to be valid also for solutes other that benzyl alcohol. The shape of the *S*_*CD*_^*i*^ profile is used as the criterion, which determines when the solute uptake by the bilayer is stopped: the transfer of the solute into the bilayer aggregate is terminated when the chain adopts the so-called limiting *S*_*CD*_^*i*^ profile. As can be seen from Table 2, the average cross-sectional area *a*_*CH*_^*SL*^ corresponding to the limiting *S*_*CD*_^*i*^ profile, *i.e.*, corresponding to the solubility limit *R*_*S*_^*SL*^, attains values from 41.76 to 45.33 Å^2^. It has been found by Gruen^42^ that free energy of −(CH_2_)_10_CH_3_ chains aggregated in bilayers, cylinders, and spheres is at a minimum in the average chain area range of 37 Å^2^< *a*_*CH*_ < 44 Å^2^ – such a range is consistent with the range of *a*_*CH*_^*SL*^ values found for the limiting *S*_*CD*_^*i*^ profile at the solubility limit *R*_*S*_^*SL*^ stated above. Even though free energy of the chains is only one contribution to the free energy of both (solute-free and solute-containing aggregate) systems, it is most likely an important factor governing the solute–bilayer (or the solute–aggregate) interactions. Other contributions to the free energy of solute-containing lipid aggregate originate from interaction of the headgroups, from chain-water contact, mixing of the solute with hydrocarbon chains, and solute contact interactions.

### Model of the Dependence of Surface Density on the Order Parameter: Relationship between Intrinsic Bilayer Forces and Bilayer Deformation Parameters

A volume element of a bilayer of surface area *A* and thickness *d*_*t*_ contains 2*N*_*L*_ lipid molecules. Since each lipid molecule is of cross-sectional area *a*_*L*_, the bilayer element surface area can be expressed as *A* = *N*_*L*_*a*_*L*_ and according to Cevc and Marsh,^10^ the equation for the change of the bilayer area due to expansion or compression at constant temperature may be written for the change of molecular area instead – the molecular area changes as a response to the change of the isotropic tension d*T̅* or of the bilayer surface tension dπ:
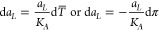
21a,b

A bilayer (bilayer membrane) can be modeled as a condensed two-dimensional gas whose molecules are hard, *i.e.,* incompressible, cylinders, which are in random thermal motion. It means that the lipid molecule cannot be compressed beyond a certain minimum value of area per molecule, the excluded area *a*_*L*_^*e*^. Therefore, from the average area per molecule *a*_*L*_, it is only the area (*a*_*L*_ – *a*_*L*_^*e*^), the excess area, that is available for compression or that can be attained by expansion of the bilayer surface. According to eq 21, the change of the area per molecule *da*_*L*_ upon compression or expansion is proportional to the area per molecule *a*_*L*_. After replacement of the area per molecule *a*_*L*_ by the excess area (*a*_*L*_ – *a*_*L*_^*e*^), we obtain an equation according to which the change of the area per molecule *da*_*L*_ with tension (pressure) d*T̅* (dπ) is proportional to the excess area (*a*_*L*_ – *a*_*L*_^*e*^):
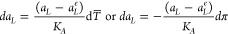
22a,b

This description of a real bilayer system accounts for the repulsive forces acting between the molecules but does not include the effect of the attractive ones.

Similarly, according Cevc and Marsh^10^ and due to volumetric incompressibility of the bilayer, the change of bilayer thickness can be written in the form
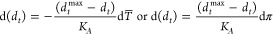
23a,b

which means that the change in the bilayer thickness upon compression or expansion is proportional to the difference (*d*_*t*_^*max*^ – *d*_*t*_), where *d*_*t*_^*max*^ is the maximum bilayer thickness corresponding to the most compressed state of the bilayer, *i.e.*, the state, when the area of lipid molecule is at its minimum *a*_*L*_^*e*^. After expressing the bilayer thickness *d*_*t*_ in terms of the average area per molecule *a*_*L*_ and the polar region thickness *d*_*p*_, eq 10, we obtain from eq 23a,b
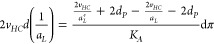
24aand:
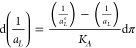
24bwhich describes the behavior of surface density (1/*a*_*L*_). The change in surface density d(1/*a*_*L*_) with pressure is proportional to difference , where (1/*a*_*L*_^*e*^) is the maximum surface density of lipid molecules.

In the treatment so far, the quantity of the average order parameter ⟨*S*⟩ has represented the ordering state of the hydrocarbon chains. Another possible way of interpreting ⟨*S*⟩ will be attempted. Consider the impact of the lateral surface pressure π on the structure of the lipid molecules in a monolayer. Depending on the surface pressure applied, the surface monolayers can adopt gaseous, liquid-like, and solid-like states.^48^ An increase of the surface pressure π leads to condensation of a sparsely covered surface consisting of less ordered molecules, *i.e.*, of a gas-like film, to a liquid-like film with progressively higher surface density (1/*a*_*L*_) and more ordered hydrocarbon chains. When the monolayers are highly compressed, they undergo a transition to a solid-like state, which is characterized by immobile adsorbed molecules that occupy the minimum area and the hydrocarbon chains have maximum order. It can be seen from this description that the two physical quantities, the surface pressure π and the average order parameter ⟨*S*⟩, can be related: the minimum order corresponds to the gaseous monolayer with independently moving adsorbed molecules, *i.e.*, the monolayer at the lowest pressure. The molecules are more ordered when the pressure is increased and maximum order is achieved when the monolayer is highly compressed. Thus, we can assume that the average order parameter can represent not only the ordering state of the hydrocarbon chain but can also represent the surface pressure in the monolayer. A similar conclusion can be drawn for the bilayer. However, apart from the bilayer surface pressure π*,* in multilamellar stacks of bilayers, there are also interbilayer stresses present – therefore, the order parameter may represent the total pressure balance in the bilayer.

It can be expected from the relation described above between the average order parameter ⟨*S*⟩ and the pressure balance in the bilayer that the change of the surface density (1/*a*_*L*_) upon ordering and disordering of the lipid molecules is proportional to the difference  in the same way as the change of the surface density d(1/*a*_*L*_) is proportional to this difference upon compression or expansion of the bilayer, eq 24b. Therefore
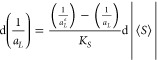
25

Under the assumption that for ⟨*S*⟩ = 0, the surface density is (1/*a*_*L*_^*max*^) = 0.002 Å^2^ (estimated in Results), the following function, which expresses the dependence of the surface density (1/*a*_*L*_) on the average order parameter ⟨*S*⟩, is the solution of eq 25:
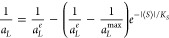
26which is the same functional form as the solution of the empirical equation defined by eq 5. Considering that the area per molecule is twice the cross-sectional area of hydrocarbon chain (*a*_*L*_ = 2*a*_*CH*_), the surface density of the hydrocarbon chains depends on the average order parameter:
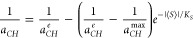
27

It means, that with increasing modulus of the order parameter (*i.e.*, with increasing order of the molecules), the surface density of the hydrocarbon chains increases until it reaches the maximum value of (1/*a*_*L*_^*e*^). It is possible to see that eqs 25, 26, and 27 derived above under the assumption that the order parameter is a representation of the balance of pressures within the bilayer and by replacing such pressures by the order parameter in the equations for bilayer area or thickness change under compression or expansion are the same as eqs 5, 6, and 7 obtained for the mathematical model of bilayers that was derived in section Results starting from two slightly different assumptions: (1) empirically proven relationship between surface density of chains and the order parameter; (2) analogy with other phenomena related to concentration. This means that both approaches led to the same mathematical and physical description of the bilayer structure as a function of order parameter. Such an agreement enables us to say that the notion that the average order parameter is a representation of the pressure balance in the bilayer indicates also the physical meaning of the parameter *K*_*S*_: *K*_*S*_ is a dimensionless parameter that represents either compressibility or some other elasticity characteristic of the bilayer.

### Effect of Hydration on the Balance of Bilayer Forces

Lipid and/or bilayer hydration is determined mainly by interactions of water molecules with lipid headgroups. The addition of water to dry lipid causes a general loosening of the lipid packing density, which is particularly large when adsorbed water molecules disrupt the pre-existing headgroup–headgroup bonds, as is the case with phosphatidylcholines.^32^ Indeed, upon increasing the hydration, an overall decrease in the segmental order parameters along the full length of the lipid chain was documented (Figure S7), while the hydrated lipid molecules adopted a preferred conformation and this conformation remained similar to the conformation in the crystal.^18,37^

The loosening of the packing of lipid molecules is accompanied by the increase of the average cross-sectional chain area *a*_*CH*_ (*i.e.*, by the decrease of the surface density of chains (1/*a*_*CH*_)) and by the decreases in the order of chain segments in solute-free DMPC bilayer (Table S2). According to the relationship between intrinsic bilayer forces and the deformation parameters^10^ that was transformed into eq 23, the change in the chain area *a*_*CH*_ (or in the surface density (1/*a*_*CH*_)) corresponds to the change in the bilayer surface pressure dπ (or the change in the isotropic tension d*T̅*). For an isolated bilayer, if there are no external forces applied to the bilayer (*i.e.*, the isotropic tension *T̅* = 0 in the equation for the mechanochemical equilibrium of the bilayer), the bilayer surface pressure π is determined by the interfacial free-energy density of the hydrophobic interaction γ.^10,49,50^ The situation is different in systems that consist of mutually interacting bilayers. The isolated and the interacting bilayers can have different properties. Bilayers within multilayers can be considered to be isolated only when the two following criteria are met:^51^ (1) the energy of interactions between bilayers is small compared to their thermal energy, *kT*; (2) no changes in a bilayer’s properties can be detected as the distance from its neighbor changes. Experimental measurements as well as theoretical predictions indicate that bilayers separated by about 1 to 200 Å, depending on their molecular components, can be considered to be isolated. Thus, if bilayers within multilayers are not isolated – in our systems, the bilayer separation expressed as *d*_*W*_ varied from ≈6 Å at hydration *R*_*W*_ = 8 to ≈19 Å at hydration *R*_*W*_ = 24 – their interactions across the interjacent water layer must be taken into consideration. These interactions produce various attractive and repulsive forces between the bilayers^52^ and according to Cevc and Marsh,^10^ even if the forces act in the direction perpendicular to the bilayer surface producing thus normal stress σ_*t*_, then due to the volumetric incompressibility of the bilayer, this normal stress σ_*t*_ is transformed into the isotropic tension *T̅* acting within the plane of the bilayer. The bilayer surface pressure π in a bilayer multilayer system at given equilibrium separation *d*_*W*_ (*i.e.*, at given hydration *R*_*W*_ and chain area *a*_*CH*_) is determined not only by the interfacial tension at the hydrophobic/hydrophilic (hydrocarbon/water) interface γ but also by the isotropic tension *T̅* present in the bilayer due to the interbilayer interactions. The distribution of lateral forces on solute molecules embedded at different depths in the bilayer depends upon how the balance of the stresses is achieved.^50^ Due to the mentioned impact of hydration on the equilibrium of the stresses acting within the bilayer, it can be expected that hydration will affect also the interaction of the bilayer with a solute. This was manifested in the hydration dependence of the value of *k*_*W*_ (the gradient of the dependence of ε-value on *R*_*S*_) as well as in the hydration dependence of the distribution of benzyl alcohol in the bilayer represented by the fraction of benzyl alcohol in sites III and IV, *X*_*S*_^*HC*^, and in different qualitative changes of the segmental order parameter profiles at high and at low hydration.

## Conclusions

A study of structural changes in 1,2-dimyristoyl-*sn-*glycero-3-phosphatidylcholine/water bilayers was performed to assess the effect of small amphiphilic molecule – an anesthetic benzyl alcohol – on the physical properties of the lipid bilayers that served as model biomembranes. The binding sites of benzyl alcohol within the bilayer were determined – the solute was shown to partition between several sites both in the headgroup and in the hydrocarbon region; such dynamic equilibrium was shown to be dependent on the level of hydration and temperature. The limits of solubility were examined and they are likely to coincide with the limiting values of ordering along the hydrocarbon chain. Our findings have possibly more general implications for dissolution of other small-molecule amphiphilic solutes since the binding of benzyl alcohol is most likely affected by the overall balance of intrabilayer and interbilayer forces in combination with the elastic properties of the bilayer.

A model of the dependence of surface density of lipid chains on the chain segment order parameter has been developed – an empirical mathematical model based on experimental data has been derived and it has been proposed to represent a relationship between intrinsic bilayer forces and bilayer deformation characteristics. Again, it might be proven in the future that the model is of more general significance.

## Supporting Information Available

Evaluation of bilayer structural parameters, parameters of macro-and mesostructure, additional ^31^P NMR spectra, solubility limits, effect of hydration on hydrocarbon chain ordering, effect of benzyl alcohol on hydrocarbon chain ordering at low hydration, quantification of benzyl alcohol distribution, limiting behavior of the lipid chains (PDF)

## ABBREVIATIONS AND SELECTED SYMBOLS

*A*: Bilayer element area
⟨*A*⟩: Average area per lipid molecule
*a*_*CH*_: Average cross-sectional area of a hydrocarbon chain
*a*_*CH*_^*e*^: Excluded area of hydrocarbon chain
*a*_*CH*_^*e*, *r*^: Excluded area of hydrocarbon chain determined experimentally
*a*_*CH*_^*max*^: Maximum area of hydrocarbon chain
*a*_*CH*_^*SL*^: Average cross-sectional area of a hydrocarbon chain at solubility limit
(1/*a*_*CH*_): Surface density of hydrocarbon chains, inverse area of a hydrocarbon chain
(1/*a*_*CH*_^*e*^): Maximum surface density of hydrocarbon chains
(1/*a*_*CH*_^*e*, *r*^): Maximum surface density of hydrocarbon chains obtained experimentally
(1/*a*_*CH*_^*max*^): Minimum surface density of hydrocarbon chains
*a*_*L*_: Average cross-sectional area of lipid molecule
(1/*a*_*L*_): Surface density of lipid molecules, inverse area of a lipid molecule
(1/*a*_*L*_^*e*^): Maximum surface density of lipid molecules
*a*_*L*_^*e*^: Excluded area of lipid molecule
*a*_*L*_^0^: Area per phospholipid molecule in crystal
*a*_*L*_^*max*^: Maximum average cross-sectional area of lipid molecule
(1/*a*_*L*_^*max*^): Minimum surface density of lipid molecules
*C*: Concentration of oxygen in water in time t or capacity
*C*_*S*_: Maximum concentration of oxygen in water
*D*: Lamellar repeat distance
⟨*d*⟩: Mean distance along the bilayer normal per one CD_2_ group
*d*_*CH*_: Thickness of the hydrocarbon region of a bilayer
*d*_*CH*_^*max*^: Maximum thickness of the hydrocarbon region of a bilayer
*d*_*CH*_^*min*^: Minimum thickness of the hydrocarbon region of a bilayer
*d*: Length per CD_2_ group along a hydrocarbon chain
*d*_*P*_: Length of the polar region of a phoshatidylcholine molecule, thickness of the headgroup region
*d*_*t*_: Bilayer thickness
*d*_*t*_^*max*^: Maximum thickness of a bilayer corresponding to (a) all solute molecules placed in the hydrocarbon region; (b) the most compressed state of the bilayer (to the excluded area of lipid molecule, *a*_*L*_^*e*^)
*d*_*t*_^*min*^: Minimum thickness of a bilayer
*d*_*W*_^*max*^: Maximum thickness of water layer
*d*_*W*_^*min*^: Minimum thickness of water layer
CP: Curvature point
DP: Dispersion point
DMPC: Dimyristoylphosphatidylcholine
DPPC: Dipalmitoylphosphatidylcholine
FID: Free induction decay
PC: Phosphatidylcholine
NMR: Nuclear magnetic resonance
PTFE: Polytetrafluoroethylene
*R*_*i*_: Mole ratio of the *i*-th component to lipid, *i* = W, S
*R*_*S*_^*SL*^: Solubility limit of solute in a bilayer
*R*_*W*_^*CP*^: Hydration corresponding to the curvature point
*R*_*W*_^*DP*^: Hydration corresponding to the dispersion point
*R*_*W*_^*P*^: Number of water molecules in the polar headgroup region of bilayer per one lipid molecule
*R*_*W*_^*SL*^: Hydration corresponding to the swelling limit
*R*_*W*_^*SP*^: Hydration corresponding to the saturation point
⟨*S*⟩: Average order parameter
*S*_*CD*_: C–D bond order parameter
*S*_*CD*_^*i*^: C–D bond order parameter of the segment in the position i
*S*_*CD*_^*P*^: C–D bond order parameter in the plateau region
⟨*S*^*SL*^⟩: Average order parameter at solubility limit found by regression analysis
⟨*S*_*r*_^*SL*^⟩: Average order parameter at solubility limit obtained experimentally
⟨*S*^0^⟩: Average order parameter in solute-free bilayer found by regression analysis
⟨*S*_*r*_^0^⟩: Average order parameter in solute-free bilayer obtained experimentally
SL: Swelling limit
*sn*: Stereospecific numbering
SP: Saturation point
