# Supplementary material for: Structural and Dynamical Response of Lipid Bilayers to Solvation of an Amphiphilic Anesthetic
Source: J Phys Chem B. 2025 Jan 24;129(5):1563–85. doi: 10.1021/acs.jpcb.4c05176 (PMC11808788; doi:10.1021/acs.jpcb.4c05176)
Supplement: Supplementary file 1 — jp4c05176_si_001.pdf [file jp4c05176_si_001.pdf]

Supporting Information:

Structural and Dynamical Response of Lipid Bilayers to Solvation of an Amphiphilic Anaesthetic.

Adriana Šturcová*

Department of Polymers for Electronics and Photonics, Institute of Macromolecular Chemistry, Czech Academy of Sciences, Heyrovského nám. 2, 162 00, Prague 6, Czech Republic

sturcova@imc.cas.cz

Evaluation of Bilayer Structural Parameters from Lamellar Repeat Distance D Determined by X-ray Diffraction

1 Determination of the Average Surface Area per Lipid Molecule $\langle A \rangle$

Consider a lipid bilayer element of surface area *A* such as shown in Figure S1. The volume element $\left(A\frac{D}{2}\right)$ contains N_L, N_W, N_S molecules of lipid, water and solute, respectively:

$$A = \frac{2}{N_A D}\sum_i N_i V_i \qquad \text{for } i = \text{L, W, S} \qquad \text{Eq. (S1)}$$

where N_A is Avogadro constant and V_i is the partial molar volume of the component *i* assumed to be equal to molar volume,[1–2] *D* is the lamellar repeat distance.

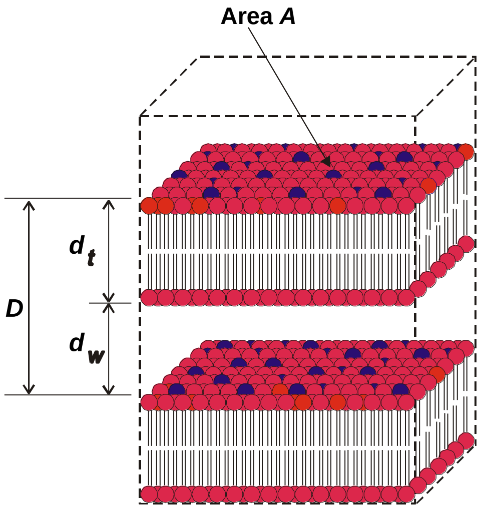

Figure S1. *Element of a lipid bilayer in lamellar phase of surface area A showing the lamellar repeat distance D, the bilayer thickness* d_t *and the interbilayer water layer thickness* d_W*. Reprinted with permission from ref. 13. Copyright 2000 University of Leeds and Šturcová.*

Average surface area per lipid at the bilayer/water interface is defined as:

$$\langle A \rangle = \frac{A}{N_L} = \frac{2}{N_A D}\sum_i R_i V_i \qquad \text{Eq. (S2)}$$

where R_i is the mole ratio of the *i*-th component to lipid. In a bilayer containing no solute (the solute-free system), Figure S2, the average area per lipid is equal to the average lipid cross-sectional area a_L, which is twice the average cross-sectional area down the hydrocarbon chain a_{CH}:

$$\langle A \rangle = a_L = 2a_{CH} \qquad \text{Eq. (S3)}$$

For a bilayer containing R_S molecules of solute per one lipid molecule, Figure S3, the average area $\langle A \rangle$:

$$\langle A \rangle = a_L + a_S R_S \quad \text{Eq. (S4)}$$

while a_S is the average surface area of the solute molecule.

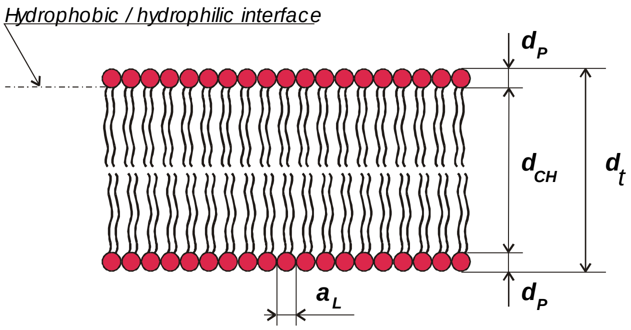

Figure S2. *Lipid bilayer dimensions: the thickness of the polar headgroup region (d_P) the thickness of the hydrocarbon region (d_{CH}) and the thickness of the bilayer (d_t) lipid cross-sectional area (a_L). Reprinted with permission from ref. 13. Copyright 2000 University of Leeds and Šturcová.*

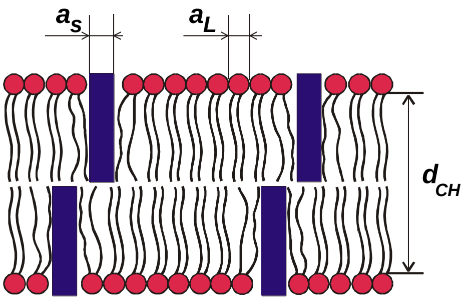

Figure S3. *A lipid bilayer containing solute of cross-sectional area a_S, the lipid cross-sectional area is a_L. Reprinted with permission from ref. 13. Copyright 2000 University of Leeds and Šturcová.*

2 Determination of Bilayer Thickness d_t

Luzzati[3] showed that in the absence of a solute d_t can be estimated as:

$$d_t = D\varphi_L \quad \text{Eq. (S5)}$$

where φ_L is the volume fraction of the lipid defined as:

$$\varphi_L = \frac{V_L X_L}{\sum_i V_i X_i} \quad \text{Eq. (S6)}$$

where X_i is the mole fraction of component *i*. However, the relationship, Eq. (S5), is valid only if a negligible amount of water is present in polar headgroup region of the bilayer, which is true if $\langle A \rangle$ is approximately independent of hydration. With regard to the bilayer phases of symmetric diacyl–phosphatidylcholines, X-ray diffraction studies[4–6] suggest that this is only true in the gel and subgel phases containing no solute.

If presence of water and a solute in the bilayer is considered, the bilayer thickness is:[7]

$$d_t = D\frac{(V_L + R_W^P V_W + R_S V_S)}{\sum_i R_i V_i} \quad \text{Eq. (S7)}$$

where R_W^P is the number of water molecules present in the polar region of the bilayer per one lipid molecule and is defined:[7]

$$R_W^P = \frac{N_A \Delta v_P}{V_W} \quad \text{Eq. (S8)}$$

where Δv_P is the volume per lipid molecule in the polar region of the bilayer in whom water can reside.

The volume of the polar (or hydrophilic) region of the lipid, v_P, which includes the glycerol backbone and the headgroup, in the case of phosphoglycerides is given as:

$$v_P = a_L^P d_P \quad \text{Eq. (S9)}$$

where d_P is the length of the polar region of the lipid molecule perpendicular to the bilayer surface.

For bilayers containing no solute or those where the solute is highly hydrophobic and hence, confined to the hydrocarbon chain region of the bilayer:[7]

$$\Delta v_P = (\langle A \rangle - a_L^P) d_P \qquad \text{Eq. (S10)}$$

If the solute preferentially resides in the polar region of the bilayer:[7]

$$\Delta v_P = (\langle A \rangle - a_L^P) d_P - R_S \frac{V_S}{N_A} \qquad \text{Eq. (S11)}$$

Nagle and Wilkinson[8] have estimated that the value of v_P for phosphatidylcholines is 344 Å^3 and is approximately independent of bilayer phase concerned. The value of a_L^P is estimated to be approximately 45 Å^2 in the L_α, $P_{\beta'}$, $L_{\beta'}$ and $L_{\sigma'}$ phases, based on X-ray diffraction studies.[5–7] Using these values in Eq. (S9) gives $d_P \approx 7.6$ Å for phosphatidylcholine bilayers.

3 Determination of the Thickness of the Hydrocarbon Region of the Bilayer d_{CH}

d_{CH} can be determined directly by a determination of the volume fraction of the hydrocarbon region, *i.e.* for bilayers containing no solute or one which is not predominantly located in the hydrocarbon core of the bilayer:[7]

$$d_{CH} = D \frac{N_A v_{HC}}{\sum_i R_i V_i} \qquad \text{Eq. (S12)}$$

where v_{HC} is the volume of the hydrocarbon portion of the lipid molecule.

For bilayers containing hydrophobic solutes located in the hydrocarbon region of the bilayer:[7]

$$d_{CH} = D \frac{N_A v_{HC} + R_S V_S}{\sum_i R_i V_i} \qquad \text{Eq. (S13)}$$

The thickness of the hydrocarbon region of the bilayer can be also defined by a relationship:

$$d_t = d_{CH} + 2 d_P \qquad \text{Eq. (S14)}$$

where d_P represents the thickness of the polar headgroup region assumed to be identical with the length of the polar region of the lipid molecule perpendicular to the bilayer surface. Therefore, if d_t has been calculated, d_{CH} can be determined using Eq. (S14).

4 Determination of the Water Layer Thickness d_W

If the bilayer thickness d_t has been calculated, d_W can be determined using Eq. (S15):

$$D = d_t + d_W \qquad \text{Eq. (S15)}$$

If d_t is unknown, the water layer thickness can be determined as:[7]

$$d_W = D \frac{(R_W - R_W^P) V_W}{\sum_i R_i V_i} \qquad \text{Eq. (S16)}$$

Macro- and meso-structure of DMPC/water bilayer systems

Bilayer hydration R_W is expressed as molar ratio of water to lipid or as the number of water molecules per one lipid molecule. The saturation point (R_W^{SP}), the curvature point (R_W^{CP}), the swelling limit (R_W^{SL}) and the dispersion point (R_W^{DP}) are levels of hydration corresponding to the macrostructure transition points that are related to the underlying changes in the mesostructure of lipid bilayers (*i.e.* increase in headgroup hydration and concomitant bilayer expansion, increase in interbilayer water thickness, formation of polyhedral liposomes and finally formation of curved liposomes) and their determination is described in the main text section Results.

Table S1. *Macro- and mesostructure transition points of DMPC/water/benzyl alcohol bilayers in L_α phase. Temperature 313.15 K. Taken from Turnbull.[7] Reprinted with permission from ref. 13. Copyright 2000 University of Leeds and Šturcová.*

R_S	R_W^{SP}	R_W^{CP}	R_W^{SL}	R_W^{DP}	
0.0	9.4±1.0	25.0±2.5	36.0±3.0	-	*a*
	-	24.4±1.8	-	50.1±4.0	*b*
0.20±0.03	9.3±1.2	26.2±1.5	39.5±4.5	-	*a*
	-	27.2±2.0	-	52.7±3.5	*b*
0.40±0.04	10.4±1.4	30.0±2.6	42.0±4.5	-	*a*
	-	-	-	-	*b*
0.80±0.05	NM	32.5±3.0	51.5±7.0	-	*a*
	-	38.1±4.0	-	62.5±5.5	*b*
1.20±0.06	-	46.1±4.0	-	77.5±5.5	*b*

(a) Determined by low-angle diffraction measurements. (b) Determined by turbidity measurements. NM means non-measurable.

Composition and structural parameters of DMPC/water bilayers

Table S2. *The composition, the average cross-sectional area of the chain (a_{CH}) and the order parameters of DMPC/water bilayers at temperature 313 K. Reprinted with permission from ref. 13. Copyright 2000 University of Leeds and Šturcová.*

**Hydration R_W**	$\lvert\langle S\rangle\rvert$ where $\langle S\rangle = \frac{1}{11}\sum_{i=3}^{n=13} S_{CD}^{i}$	$\lvert S_{CD}^{P}\rvert$	a_{CH} [Å²]	**Reference**
5	0.256	--	25.2	*a*
5.8	0.223 ± 0.008	0.255	26.15 ± 0.40	
7	0.239 ± 0.005	0.259	26.65 ± 0.45	*b*
8.9	0.209 ± 0.009	0.239	27.70 ± 0.40	*b*
9.9	0.201 ± 0.006	0.222	28.15 ± 0.35	*b*
11.6	0.187 ± 0.009	0.207	28.50 ± 0.35	*b*
14.9	0.184 ± 0.006	0.204	29.80 ± 0.50	*b*
17	0.184 ± 0.009	0.208	29.25 ± 0.40	*b*
17.8	0.174 ± 0.010	0.192	29.90 ± 0.45	*b*
20	0.171 ± 0.008	0.191	30.35 ± 0.55	*b*
22.9	0.170 ± 0.009	0.189	30.86 ± 0.55	*b*
25.4	0.162 ± 0.008	0.179	31.05 ± 0.55	*b*
20	0.152 ± 0.005	0.182	31.60 ± 0.45	*c*
20	0.144 ± 0.005	0.173	32.30 ± 0.39	*c*

(a) Boden et al. (1991);[1] (b) Data obtained at SOMS Centre laboratory (University of Leeds, United Kingdom) by Dr. P. Turnbull; (c) This work.

Hydration dependence of solubility limit.

^{31}P NMR spectra obtained on DMPC bilayers at benzyl alcohol concentration R_S of 3.0 and temperature 313 K at various hydrations are presented in Figure S4. Solubility limit R_S^{SL} was found to be equal to 3.0 for hydration range from 13 to 25 water to lipid mole ratio (see also Figure S5). Solubility limit R_S^{SL} was determined to be 2.5 benzyl alcohol to lipid mole ratio for hydrations $8 \leq R_W \leq 10$ and to be 3.5 benzyl alcohol to lipid mole ratio at R_W = 40 at temperature 313 K (Figure S5 – black symbols). These results indicate that the maximum concentration of benzyl alcohol that can be accommodated by a DMPC bilayer is hydration dependent at temperature 313 K – R_S^{SL} increases as hydration is increased.

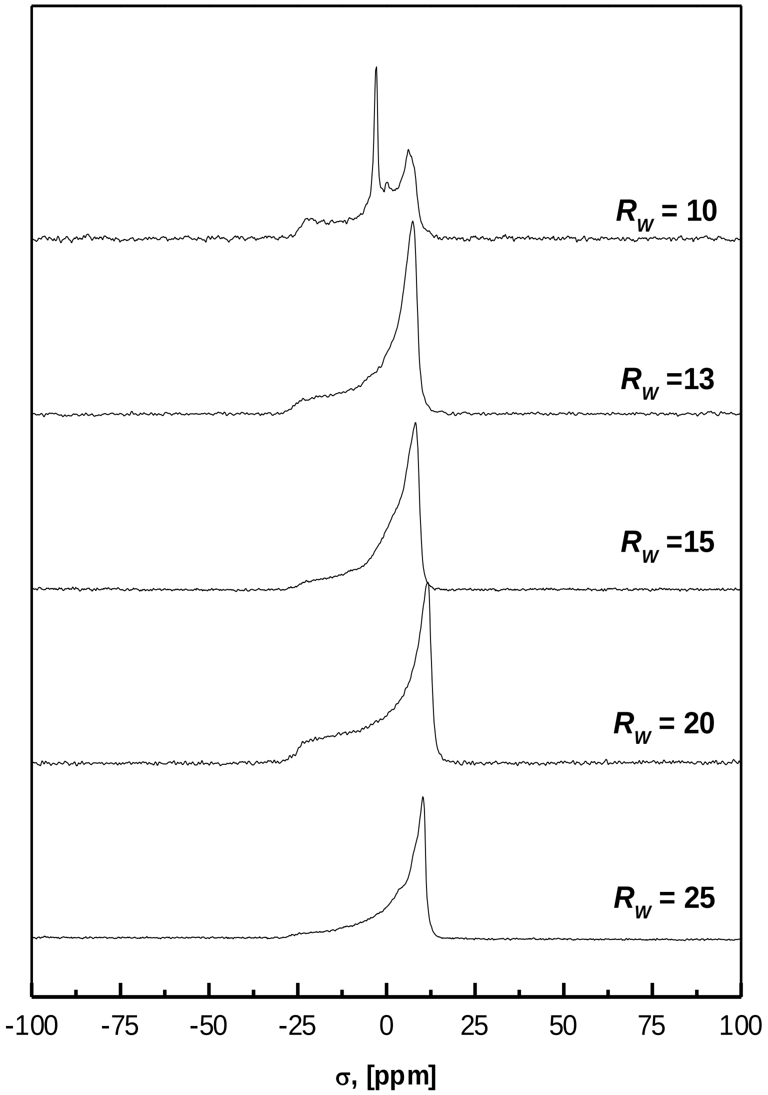

Figure S4. *Proton-decoupled* ^{31}*P NMR spectra of DMPC bilayers containing benzyl alcohol,* R_S *= 3.0, at various hydration* R_W*; temperature 313 K. Reprinted with permission from ref. 13. Copyright 2000 University of Leeds and Šturcová.*

Dependence of solubility limit on hydration and on temperature.

A graph of the dependence of R_S^{SL} on R_W has been obtained from phase diagrams of Turnbull[7] for temperature 298 K and the boundary between L_α and (L_α+BZA) phase lies at R_S = 3.0 for all the hydrations studied in that work (R_W of 17, 23, 40, 55 and 67) – red line and symbols in Figure S5. Comparison of the two boundaries for hydration R_W = 40 indicates that solubility limit R_S^{SL} increases from 3.0 to 3.5 as the temperature is raised from 298 K to 313 K.

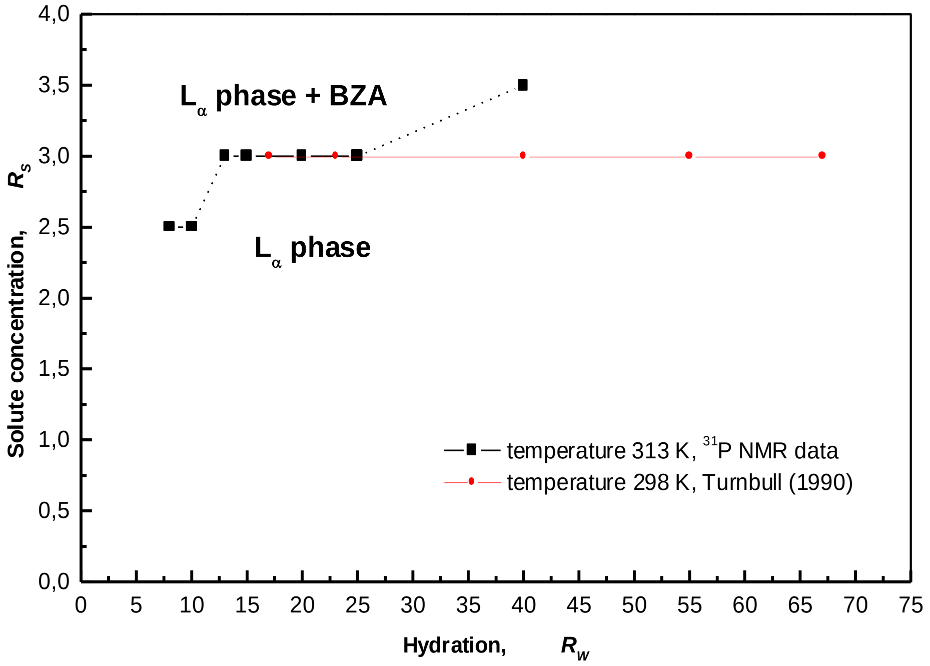

Figure S5. *Boundary between L_α phase and (L_α + BZA) phase (a) determined by ^{31}P NMR spectroscopy at temperature 313 K (black symbols and line); (b) determined by calorimetry and X-ray diffraction at temperature 298 K (red symbols and line; data taken from Turnbull[7]). The boundary at given hydration R_W and temperature is consistent with the maximum concentration of benzyl alcohol – the solubility limit R_S^{SL}, that can be attained in the bilayer without benzyl alcohol separating into the excess phase. Reprinted with permission from ref. 13. Copyright 2000 University of Leeds and Šturcová.*

Structural Parameters of DMPC Bilayer at Limited Hydration as Determined by X-ray Diffraction

Table S3. *Parameters of the mesoscopic structure of DMPC/water/benzyl alcohol bilayers at high hydration (R_W = 23), temperature 313.15 K at stated benzyl alcohol concentration (R_S). Reprinted with permission from ref. 13. Copyright 2000 University of Leeds and Šturcová.*

R_S	D [Å]	$\langle A \rangle$ [Å^2]	d_{CH}^{max} [Å]	d_{CH}^{min} [Å]	d_W^{max} [Å]	d_W^{min} [Å]
0.0	57.8 ± 2.4	62.2 ± 2.6	24.7 ± 1.0	24.7 ± 1.0	17.9 ± 1.4	17.9 ± 1.4
0.2	56.3 ± 2.3	65.1 ± 2.6	24.6 ± 1.0	23.6 ± 0.9	17.5 ± 1.3	16.5 ± 1.3
0.4	54.8 ± 2.1	68.2 ± 2.7	24.5 ± 1.0	22.5 ± 0.9	17.1 ± 1.3	15.1 ± 1.2
0.6	53.1 ± 2.0	71.6 ± 2.7	24.3 ± 0.9	21.4 ± 0.8	16.5 ± 1.2	13.6 ± 1.1
0.8	51.7 ± 1.9	74.9 ± 2.8	24.2 ± 0.9	20.5 ± 0.8	16.0 ± 1.1	12.3 ± 1.0
1.0	50.2 ± 1.8	78.5 ± 2.8	23.9 ± 0.9	19.5 ± 0.7	15.5 ± 1.1	11.1 ± 0.9
1.2	48.9 ± 1.7	82.0 ± 2.9	23.7 ± 0.8	18.7 ± 0.7	15.0 ± 1.1	10.0 ± 0.9
1.4	47.6 ± 1.6	85.7 ± 2.9	23.5 ± 0.8	17.9 ± 0.6	14.5 ± 1.0	8.9 ± 0.8
1.5	47.0 ± 1.6	87.5 ± 2.9	23.4 ± 0.8	17.5 ± 0.6	14.3 ± 1.0	8.4 ± 0.8
1.6	46.5 ± 1.5	89.2 ± 3.0	23.4 ± 0.8	17.2 ± 0.6	14.1 ± 1.0	7.9 ± 0.8
1.8	45.4 ± 1.5	92.9 ± 3.0	23.2 ± 0.7	16.5 ± 0.5	13.7 ± 0.9	7.0 ± 0.7
2.0	44.5 ± 1.4	96.3 ± 3.0	23.1 ± 0.7	15.9 ± 0.5	13.4 ± 0.9	6.2 ± 0.7
2.2	43.8 ± 1.4	99.4 ± 3.1	23.0 ± 0.7	15.4 ± 0.5	13.2 ± 0.9	5.6 ± 0.6
2.4	43.1 ± 1.3	102.6 ± 3.1	23.0 ± 0.7	15.0 ± 0.5	12.9 ± 0.9	4.9 ± 0.6
2.6	42.6 ± 1.3	105.4 ± 3.2	23.0 ± 0.7	14.6 ± 0.4	12.8 ± 0.8	4.4 ± 0.6
2.8	42.3 ± 1.3	107.8 ± 3.2	23.2 ± 0.7	14.2 ± 0.4	12.9 ± 0.8	3.9 ± 0.6
3.0	42.1 ± 1.3	110.0 ± 3.3	23.3 ± 0.7	14.0 ± 0.4	12.9 ± 0.8	3.6 ± 0.6

Table S4. *Parameters of the mesoscopic structure of DMPC/water/benzyl alcohol bilayers at low hydration (R_W = 8), temperature 313.15 K at stated benzyl alcohol concentration (R_S). Reprinted with permission from ref. 13. Copyright 2000 University of Leeds and Šturcová.*

R_S	D [Å]	$\langle A \rangle$ [Å^2]	d_{CH}^{max} [Å]	d_{CH}^{min} [Å]	d_W^{max} [Å]	d_W^{min} [Å]
0.0	48.2	56.0	27.4	27.4	5.6	5.6
0.2	46.0	60.2	26.6	25.5	5.3	4.2
0.4	44.2	64.2	26.1	23.9	5.1	3.0
0.6	42.6	68.2	25.5	22.5	4.9	1.9
0.8	41.0	72.6	24.9	21.1	4.7	0.9
1.0	39.6	76.9	24.4	19.9	4.4	0.0
1.2	38.6	80.7	24.1	19.0	4.4	-0.7
1.4	37.5	84.8	23.8	18.1	4.2	-1.4
1.6	36.6	88.7	23.5	17.3	4.1	-2.1
1.8	36.0	92.1	23.4	16.7	4.2	-2.5
2.0	35.6	95.2	23.3	16.1	4.3	-3.0
2.2	35.3	98.0	23.4	15.7	4.4	-3.3
2.3	35.2	99.2	23.4	15.5	4.5	-3.4
2.5	35.1	101.3	23.6	15.1	4.8	-3.7

Determination of Parameters of Bilayer Mesoscopic Structure in the Presence of Solute

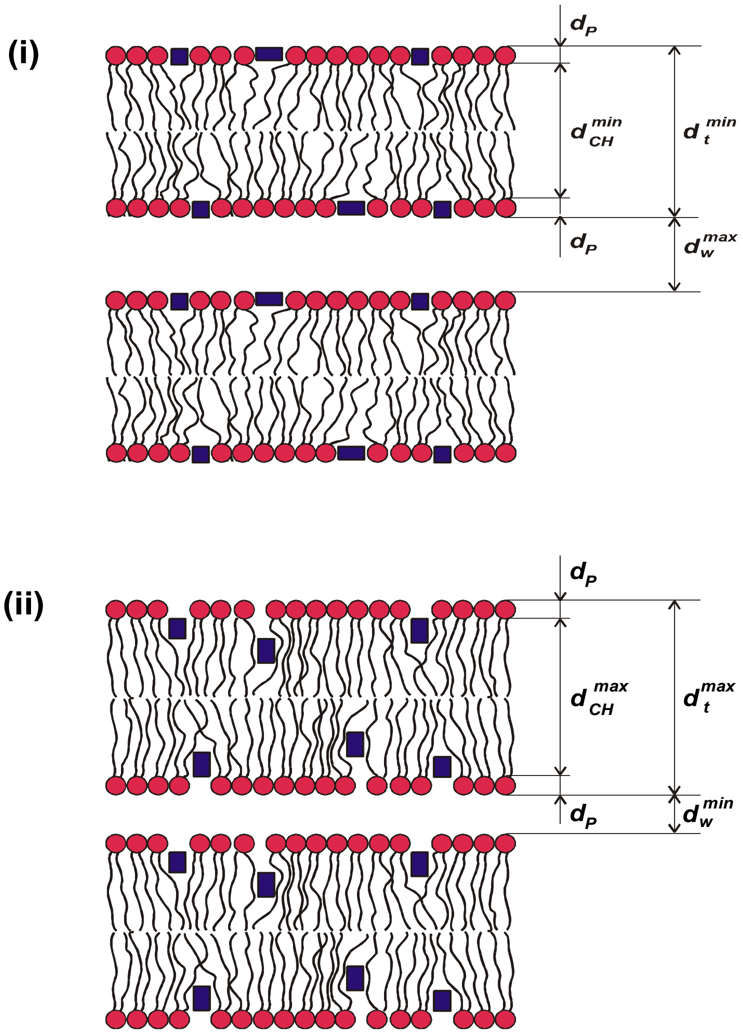

Figure S6. *Lipid bilayer containing solute when (i) all solute molecules are assumed to be placed in the headgroup region (site II), in which case the thickness of the headgroup region is d_P, the thickness of the hydrocarbon region is d_{CH}^{min}, the thickness of the bilayer is d_t^{min} and the thickness of the interbilayer water layer is d_W^{max}; (ii) all solute molecules are assumed to be in the hydrocarbon region (site III, IV and V), in this case the thickness of the headgroup region is d_P, the thickness of the hydrocarbon region is d_{CH}^{max}, the thickness of the bilayer is d_t^{max} and the thickness of the water layer is d_W^{min}. The solute is represented by a blue rectangle. Reprinted with permission from ref. 13. Copyright 2000 University of Leeds and Šturcová.*

Characterization of Lipid Molecule by NMR Spectroscopy

Effect of Hydration on Hydrocarbon Chain Ordering as Determined by Deuterium NMR

Typical ^{2}H NMR spectra are presented in Figure S7 left (*cf.* Table S2). The effect of hydration R_W on the order of the hydrocarbon chain segments of lipid molecules in solute-free DMPC bilayers at temperature 313 K can be seen in Figure S7 (right) and Figure 7 (panel a). Addition of water causes reduction of the quadrupolar splitting $\Delta\nu_Q^i$ corresponding to the CD_2 segment in position *i* in the lipid chain and consequently, causes a decrease of the segmental order parameters S_{CD}^i along the full length of the lipid chain – the order decreases in both the plateau region and the lower half of the chains. The values of S_{CD}^i were calculated from the values of $\Delta\nu_Q^i$ using Eq. (2) where S_{CD}^i was assumed to attain negative values.[9] Lipid and bilayer hydration are determined mainly by interaction of water with headgroup (the phosphate and the choline groups) and with the upper part of the apolar chain region – with the carbonyl group.[10] First two water molecules are involved in linking the phosphate groups into ribbons. Further two molecules form hydrogen bonds with the phosphate oxygen and one of the first two water molecules and at the same time form hydrogen bonds across the bilayer interface with the corresponding water molecules from the opposing bilayer.[11] Additional water molecules interact with the water molecules already present in the bilayer. This leads to the expansion of the headgroup lattice, loosening of the molecular packing and so to relieved steric constraints along the full length of the lipid molecule (Figure S7 right and Figure 7 panel a). This also gives more motional freedom to the molecules. It was shown by NMR studies[9–12] that there is a preferred conformation in lipid solutions and dispersions and the fully hydrated conformation is related to the conformation in the solid state. General decrease in the chain order with increased hydration was observed, but the shape of the order profiles of the chains stayed similar (Figure S7 right).

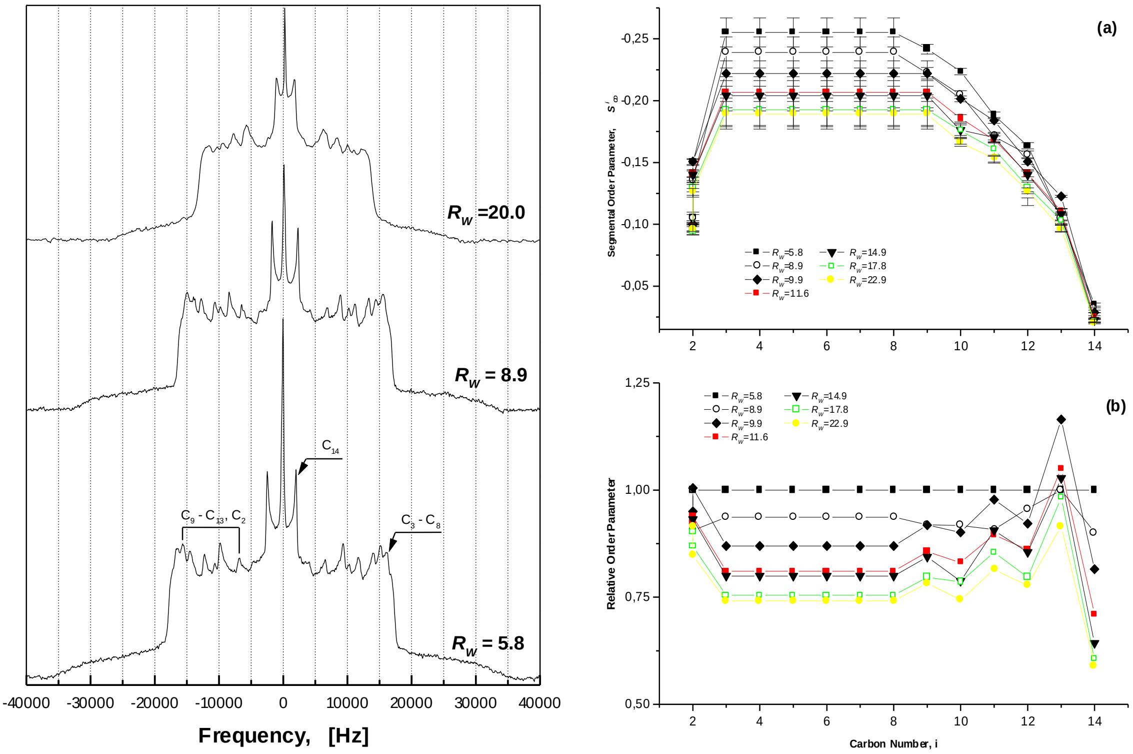

Figure S7. Left: *^{2}H NMR spectra corresponding to hydrocarbon chains in solute-free DMPC bilayers at hydration R_W 5.8, 8.9 and 20.0, temperature 313 K.* ***Right:*** *Effect of hydration on the order of the lipid chain in DMPC bilayers, temperature 313 K. (a) Segmental order parameter profiles S_{CD}^i. (b) Profiles of relative order parameter, where S_{CD}^i values corresponding to the lipid chains in bilayers at R_W = 5.8 are taken as the reference. Adapted with permission from ref. 13. Copyright 2000 University of Leeds and Šturcová.*

Effect of Benzyl Alcohol on Hydrocarbon Chain Ordering as Determined by Deuterium NMR – Low Hydration

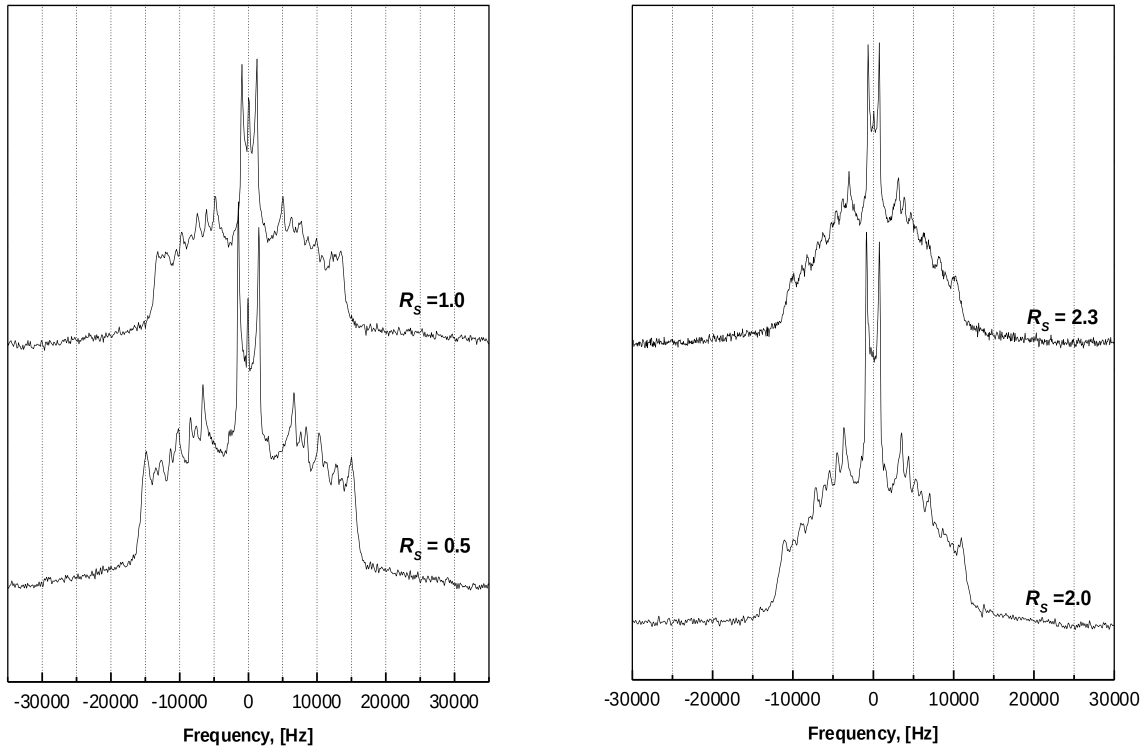

Figure S8. *^{2}H NMR spectra acquired on lipid chains in DMPC/water/benzyl alcohol system at low hydration of R_W = 8, temperature 313 K. Adapted with permission from ref. 13. Copyright 2000 University of Leeds and Šturcová.*

Quantification of Distribution of Benzyl Alcohol within the Bilayer

It has been assumed (based on X-ray diffraction data), that benzyl alcohol molecules may be distributed either into the polar headgroup region or into the non-polar hydrocarbon region of the bilayer. Comparison of the maximum and the minimum hydrocarbon region thickness determined from the X-ray data – $d_{CH}^{max}$and d_{CH}^{min}, with the hydrocarbon region thickness determined from NMR data – d_{CH}, confirmed the existence of site II and the existence of sites III and IV for accommodation of benzyl alcohol in the bilayer.

It can be assumed that a fraction X_S^{HC} from the total amount of benzyl alcohol added to the bilayer is present in the hydrocarbon region (sites III, IV and V). Then, using Eq. (S13), it is possible to express the thickness of the hydrocarbon region by the following relationship:

$$d_{CH} = D\frac{v_{HC}N_A + X_S^{HC}R_S V_S}{\sum_i R_i V_i} \qquad \text{Eq. (S17)}$$

where the lamellar repeat distance *D* is determined in an X-ray diffraction experiment. If it is assumed that benzyl alcohol does not occupy the site V, it is possible to use the hydrocarbon region thickness d_{CH} calculated from the average order parameter $\langle S \rangle$ determined in an NMR experiment (by use of Eq. (7) or a linear dependence in combination with Eq. (9)) in order to calculate fraction X_S^{HC} using Eq. (S17). In this case, X_S^{HC}represents only sites III and IV. The fraction of benzyl alcohol in the polar headgroup region (the site II) can be obtained using the condition:

$$X_S^{HC} + X_S^{P} = 1 \qquad \text{Eq. (S18)}$$

where X_S^{P} is the fraction of benzyl alcohol in site II. Such calculation was performed for both hydrations R_W=23 and R_W=8 and for the respective range of R_S values from a solvent-free bilayer to a bilayer at the corresponding solubility limit R_S^{SL}. The variation of X_S^{HC} with solute concentration R_S is shown in Figure S9.

Before the behaviour of the fraction in hydrocarbon sites X_S^{HC} can be interpreted, it is necessary to remind, that in the calculation of X_S^{HC} according to Eq. (S17), it was assumed that benzyl alcohol did not accumulate in the centre of the bilayer to form a separate layer, *i.e.* it did not occupy site V. If a layer of benzyl alcohol in the centre of the hydrocarbon region had been formed, then the real thickness of this region would be greater than the thickness d_{CH} obtained on the basis of NMR data. Further, when assessing accuracy of X_S^{HC} determination, one has to bear in mind that: (1) the calculation of d_{CH} values is an approximation based on data obtained in solute-free DMPC bilayers; (2) error of determination of the lamellar repeat distance ΔD will be reflected in X_S^{HC} calculation.

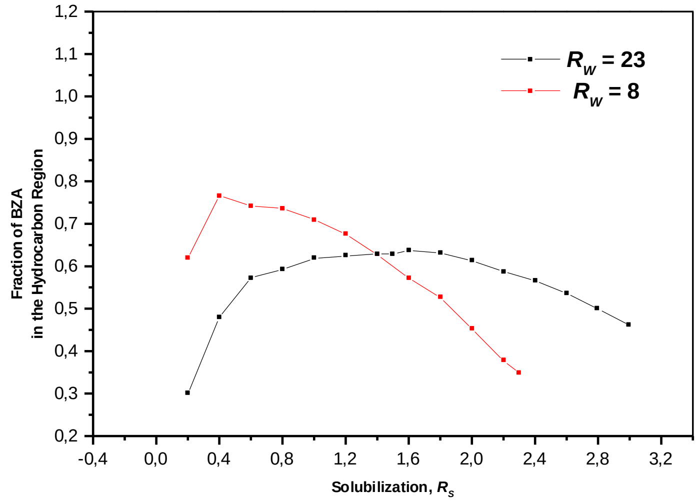

Figure S9. *Variation of fraction X_S^{HC} of benzyl alcohol located in site III and IV of DMPC bilayers from the total amount of benzyl alcohol added to the bilayers with the solute concentration R_S. The variation of X_S^{HC} with solute concentration R_S was determined for high hydration R_W = 23 (black line and symbols) and low hydration R_W = 8 (red line and symbols), temperature 313 K. Values were obtained using*

exponential fit Eq. (7). Data are reliable for $R_S \geq 1.5$ – see the following text. Reprinted with permission from ref. 13. Copyright 2000 University of Leeds and Šturcová.

A Correction of the Calculation of the Fraction of Benzyl Alcohol in Sites III and IV (X_S^{HC}) for the Error of Determination of the Lamellar Repeat Distance (ΔD)

Since the relationship for X_S^{HC} determination is known (following from Eq. (S17)):

$$X_S^{HC} = \frac{\frac{d_{CH}(V_L+V_S R_S+V_W R_W)}{D} - v_{HC} N_A}{V_S R_S} \qquad \text{Eq. (S19)}$$

it is possible to determine an error in X_S^{HC} calculation caused by an error ΔD in determination of the lamellar repeat distance:[14]

$$\left(\Delta X_S^{HC}\right)_D = \frac{\partial X_S^{HC}}{\partial D} \Delta D \qquad \text{Eq. (S20)}$$

which means that:

$$\left(\Delta X_S^{HC}\right)_D = -\frac{d_{CH} \sum_i R_i V_i}{R_S V_S D^2} \Delta D \qquad \text{Eq. (S21)}$$

Using Eq. (S21), $\left(\Delta X_S^{HC}\right)_D$ was determined for the high hydration R_W=23. and the appropriate values are shown in Figure S10 as error bars. It can be seen that the values of $\left(\Delta X_S^{HC}\right)_D$ were decreasing from ±0.86 (a 286% error) to ±0.06 (a 12% error, exponential fit) or from ±0.88 (an 110% error) to ±0.07 (a 9% error, linear fit). Thus, only the behaviour of X_S^{HC} for solute concentrations R_S of 1.5 and greater could be considered to be reliable. For the concentrations $R_S \geq 1.5$, the error $\left(\Delta X_S^{HC}\right)_D$ in X_S^{HC} determination due to the error in determination of lamellar repeat distance ΔD is not greater than ±0.12 (19% of X_S^{HC} value at R_S = 1.5, exponential fit) or ±0.13 (14% of X_S^{HC} value at R_S = 1.5, linear fit) for the high hydration, Figure S10.

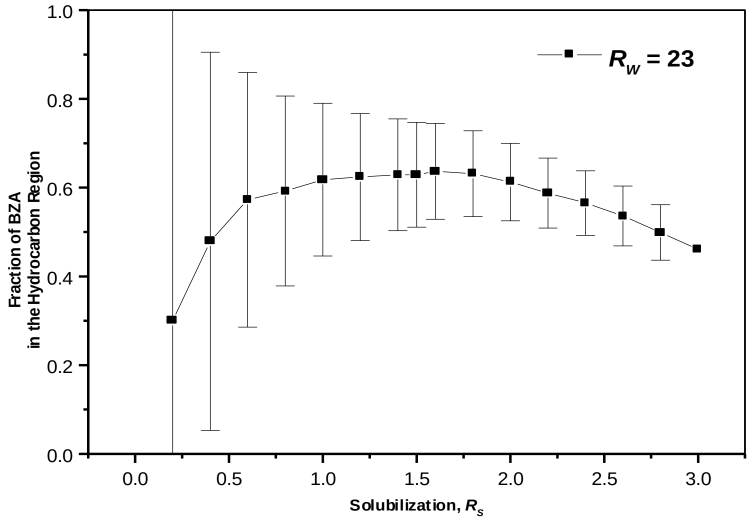

Figure S10. *Variation of the fraction of benzyl alcohol in site III and site IV (X_S^{HC}) with solute concentration R_S at high hydration R_W=23, temperature 313 K. The error bars in this graph represent the error $\left(\Delta X_S^{HC}\right)_D$ in determination of X_S^{HC} caused by the error of determination of lamellar repeat distance ΔD. These values were obtained using exponential fit according to Eq. (7). Reprinted with permission from ref. 13. Copyright 2000 University of Leeds and Šturcová.*

It can be seen from Figure S9 that X_S^{HC} is greater at the high hydration R_W=23 than at the low hydration R_W=8 for the benzyl alcohol concentrations R_S≥1.5. Values of X_S^{HC} decrease with increasing R_S for both hydrations while the decrease is steeper for the lower hydration for $R_S \geq 1.5$. The finding that X_S^{HC} varies with R_S and with R_W for $R_S \geq 1.5$ supports the conclusion made in the main text about hydration and solute concentration dependence of the population distribution of benzyl alcohol in a DMPC bilayer.

According to the condition given by Eq. (S18), the decrease of X_S^{HC} with R_S indicates that X_S^P (the fraction of benzyl alcohol molecules bound in site II) increases with R_S for $R_S \geq 1.5$. Behaviour of water layer thickness d_W (main text) indicated that water molecules were displaced from the headgroup region into the interbilayer water layer at high benzyl alcohol concentration R_S≥1.5 or R_S≥2.0 at R_W=8; and R_S≥2.5 at R_W=24. The displaced water molecules prevented the interbilayer separation from decreasing and thus prevented the increase of the interbilayer repulsive forces, which would oppose the bilayer area expansion.

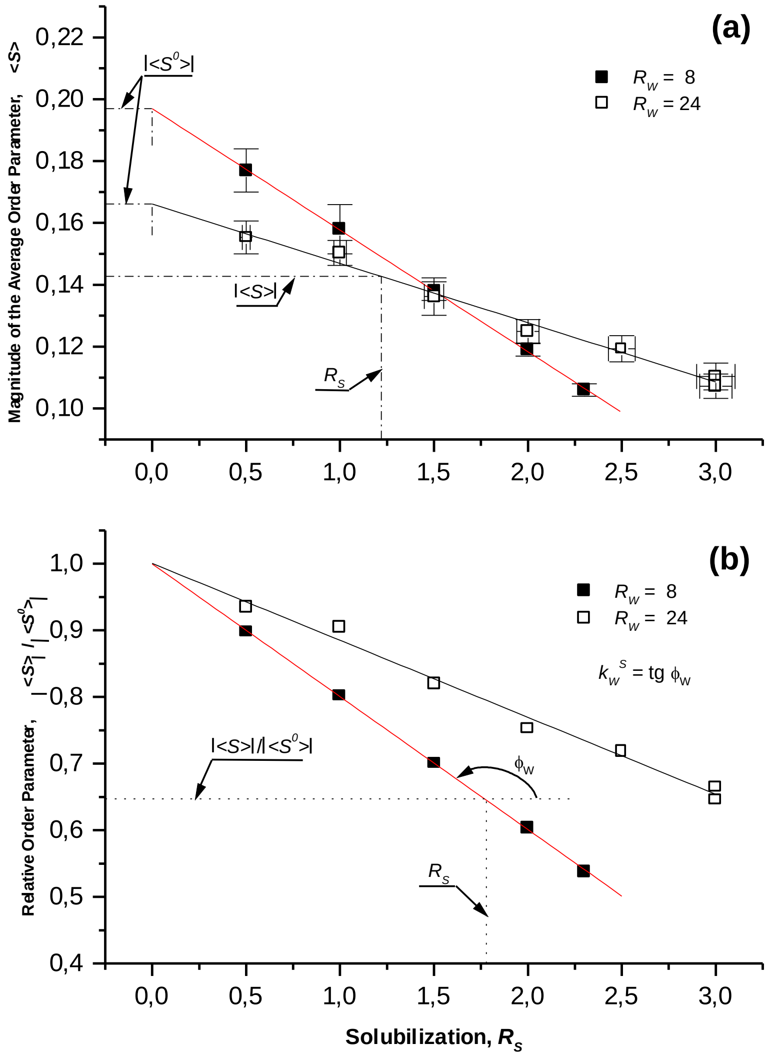

Figure S11. *(a) Variation of the modulus of the average order parameter* $|\langle S\rangle|$ *with the benzyl alcohol concentration* R_S *at the low hydration* R_W *= 8 and the high hydration* R_W *= 24. (b) Behaviour of the modulus of the relative average order parameter,* $\frac{|\langle S\rangle|}{|\langle S^0\rangle|}$*, with* R_S *at the low and the high hydration. Reprinted with permission from ref. 13. Copyright 2000 University of Leeds and Šturcová.*

Table S5. *Hydration dependence of the gradients* k_W^S[1] *Reprinted with permission from ref. 13. Copyright 2000 University of Leeds and Šturcová.*

**Hydration, R_W**	**8**	**9.9**	**14.9**	**17**	**20**	**24**
$\lvert\langle S_r^0\rangle\rvert$ [2]	0.197	0.201	0.184	0.184	0.171	0.166
$\lvert\langle S_r^{SL}\rangle\rvert$ [3]	0.106	0.108	0.108	0.108	0.108	0.107
**Solubility limit,** R_S^{SL}	2.3	2.5	3.0	3.0	3.0	3.0
**Gradient,** k_W^S	-0.201	-0.185	-0.138	-0.138	-0.123	-0.118

[1] The gradients k_W^S determined according to Eq. (14) main text. [2] The values of $\lvert\langle S_r^0\rangle\rvert$ were determined experimentally and taken from Table S2. Values at R_W = 8 and R_W = 24 are from a fit of $\langle S\rangle$ vs. R_S based on experimental data at respective hydration. [3] The value of the average order parameter at the solubility limit $\langle S_r^{SL}\rangle$ was either determined experimentally or, where value from an experiment was not available, the average of $\langle S_r^{SL}\rangle$ values presented in Table 2 was used, since it was shown that at R_S^{SL} and at the given hydration, the same limiting S_{CD}^i profile (and consequently the same $\langle S^{SL}\rangle$) is reached – see the main text.

Table S6. *Solute concentration R_S required to obtain the selected values of $\lvert\langle S\rangle\rvert$ at given hydration R_W. Reprinted with permission from ref. 13. Copyright 2000 University of Leeds and Šturcová.*

$\lvert\langle S\rangle\rvert$	***R_S*** [1]					
	***R_W* = 8**	***R_W* = 9.9**	***R_W* = 14.9**	***R_W* = 17**	***R_W* = 20**	***R_W* = 24**
0.197	0.0	0.1	-	-	-	-
0.187	0.3	0.4	-	-	-	-
0.177	0.5	0.7	0.3	0.3	-	-
0.167	0.8	0.9	0.6	0.7	0.2	-
0.158	1.0	1.2	1.0	1.1	0.6	0.4
0.155	1.1	1.3	1.1	1.2	0.8	0.6
0.138	1.5	1.7	1.7	1.9	1.6	1.4
0.136	1.5	1.8	1.8	1.9	1.7	1.5
0.119	2.0	2.2	2.4	2.6	2.5	2.4
0.109	2.2	2.5	2.8	3.0	2.9	2.9
0.106	2.3	2.6	2.9	3.2	3.1	3.0

[1] The R_S values were determined according to Eq. (20) of the main text.

Table S7. *Comparison of calculated R_S values with values from experiment. Reprinted with permission from ref. 13. Copyright 2000 University of Leeds and Šturcová.*

$\lvert\langle S\rangle\rvert$	***R_S***		$\lvert\langle S\rangle\rvert$	***R_S***	
	***R_W* = 8** [1]	***R_W* = 8** [2]		***R_W* = 24**[1]	***R_W* = 24** [2]
0.158	1.0	1.0	0.155	0.6	0.5
0.138	1.5	1.5	0.136	1.5	1.5
0.119	2.0	2.0	0.119	2.4	2.5

[1] Calculated R_S values from Table S5. [2] Values of R_S used in experiment for which the given values of the average order parameter were obtained.

REFERENCES

(1) N. Boden, S. A. Jones and F. Sixl (1991) Biochemistry 30, 2146—2155. On the use of deuterium nuclear magnetic resonance as a probe of chain packing in lipid bilayers.
(2) N. Boden, S. A. Jones and F. Sixl (1987) J. Phys. Chem. 91, 137—145. Solubilization in lyotropic liquid crystals: the concept of partial molecular surface area.
(3) V. Luzzati (1968) Biological Membranes (ed. D. Chapman) 1, Academic Press, New York, 71—123.
(4) E. Oldfield, M. Meadows, D. Rice and R. Jacobs (1978) Biochemistry 17, 2727—2739. Spectroscopic studies of specifically deuterium labeled membrane systems. Nuclear magnetic resonance investigation of the effect of cholesterol in model systems.
(5) M. J. Janiak, D. M. Small and G. G. Shipley (1979) J. Biol. Chem. 254, 6068—6078. Temperature and compositional dependence of the structure of hydrated dimyristoyl lecithin.
(6) M. J. Ruocco and G. G. Shipley (1982) Biochim. Biophys. Acta – Biomembranes, 309—320. Characterization of the sub-transition of hydrated dipalmitoylphosphatidylcholine bilayers. Kinetic, hydration and structural study.
(7) P. J. H. Turnbull (1990) Ph.D. Thesis, University of Leeds. The interaction of benzyl alcohol with lipid bilayers.
(8) J. F. Nagle and D. A. Wilkinson (1978) Biophys. J. 23, 159—175. Lecithin bilayers – density measurement and molecular interactions.
(9) J. Seelig (1977) Q. Rev. Biophys. 10, 353—418. Deuterium magnetic resonance: theory and application to lipid membranes.
(10) G. Cevc and D. Marsh (1987) Phospholipid Bilayers: Physical principles and models, John Wiley and Sons, New York.
(11) H. Hauser, I. Pascher, R. H. Pearson and S. Sundell (1981) Biochim. Biophys. Acta 650, 21—51. Preferred conformation and molecular packing of phosphatidylethanolamine and phosphatidylcholine.
(12) Seelig (1978) Biochim. Biophys. Acta – Reviews on Biomembranes 515, 105—140. ^{31}P nuclear magnetic resonance and the head group structure of phospholipids in membranes.
(13) A. Šturcová (2000) Ph.D. Thesis, SOMS Centre, School of Chemistry, University of Leeds. Studies of interactions of amphiphilic solutes with lipid bilayers.
(14) J. Skákala (1986) ES SVŠT, Bratislava, 72—77. General Metrology (in Slovak language).
